# Network pharmacology combined with untargeted metabolomics reveals the intervention mechanism and compatibility of chenpi-rougui herb pair in nonalcoholic fatty liver disease

**DOI:** 10.3389/fmolb.2025.1553162

**Published:** 2025-03-13

**Authors:** Jinlin Xu, Sen Li, Yuehui Jin, Huiwen Yao, Xu Hu, Shan Cao, Huimin Zhou

**Affiliations:** ^1^ School of Pharmacy, Hubei University of Chinese Medicine, Wuhan, Hubei, China; ^2^ Department of Pharmacy, Ezhou Central Hospital, Ezhou, Hubei, China; ^3^ Department of Pharmacy, Union Hospital, Tongji Medical College, Huazhong University of Science and Technology, Wuhan, Hubei, China; ^4^ School of Traditional Chinese Medicine, Hubei University of Chinese Medicine, Wuhan, Hubei, China; ^5^ School of Nursing, Hubei University of Chinese Medicine, Wuhan, Hubei, China

**Keywords:** chenpi-rougui herb pair, non-alcoholic fatty liver disease, untargeted metabolomics, network pharmacology analysis, synergistic effect

## Abstract

**Background:**

Chenpi (the dried mature peel of *Citrus reticulata* Blanco) and Rougui (the dried bark of *Cinnamomum cassia* Presl) are both edible and medicinal plants, which have therapeutic effects on nonalcoholic fatty liver disease (NAFLD). However, the underlying mechanisms necessitate further exploration. This study evaluated the prevention effect of Chenpi-Rougui herb pair (CRP) on NAFLD using an integrated strategy that combined network pharmacology with metabolomics.

**Methods:**

Initially, the components in CRP decoction were characterized by UPLC-QTOF-MS/MS. Subsequently, a high-fat diet induced NAFLD mouse model was used to assess the efficacy of CRP and its individual constituent, Chenpi and Rougui. Additionally, synergetic pathways and crucial targets for CRP therapy in NAFLD were identified using network pharmacology and serum metabolomics. Finally, real-time polymerase chain reaction (RT-PCR) was utilized to validate relevant genes.

**Results:**

CRP exerted a more extensive prevention effect on NAFLD mice compared to the individual herb of Chenpi and Rougui. A total of 105 compounds were characterized from CRP, which were linked to 70 potential therapeutic targets for NAFLD. Thirty-two differential metabolites were identified by metabolomics, which were co-regulated by Chenpi, Rougui and CRP. Pathways associated with the intervention of herb pair in NAFLD included energy metabolism, fatty acid metabolism, glycerophospholipid metabolism, sphingolipids metabolism, arachidonic acid metabolism, sterol and bile acid metabolism. Finally, eight targets were screened through conjoint analysis and experimental verification showed that six of them including FASN, AKT1, CASP3, F2, PTGS2 and PRKCA, could be modulated by CRP in NAFLD mice. Besides, Chenpi primarily regulated FASN, AKT1, CASP3 and PRKCA, which were associated with reducing apoptosis in hepatocytes, while Rougui exceled in regulating F2 and PTGS2, closely linked to its anti-inflammatory properties. The combination of Chenpi and Rougui resulted in a broader influence on metabolites, pathways, and primary targets compared to their individual application.

**Conclusion:**

These results provided valuable insights into the compatibility mechanism of CRP for treating NAFLD, and could also improve the value of its forthcoming application and development as a natural liver protective agent.

## 1 Introduction

Nonalcoholic fatty liver disease (NAFLD), a clinicopathological liver syndrome characterized by hepatic lipid accumulation in the absence of significant alcohol consumption or genetic disorders, has emerged as a leading chronic liver disease worldwide, affecting approximately 25.24% of adults (Chalasani et al., 2018). Its progression to steatohepatitis, fibrosis, cirrhosis, and hepatocellular carcinoma imposes substantial physical and psychological burdens on patients (Tziomalos et al., 2015; Calzadilla Bertot and Adams, 2016). Current pharmacological interventions, such as insulin sensitizers and hypolipidemic agents, exhibit limited efficacy and potential adverse effects, including weight gain, cardiovascular risks, and hepatotoxicity (Colca et al., 2023). These limitations underscore the urgent need for safer and more effective therapeutic strategies.

In recent years, medicinal foods, particularly those classified as medicine food homology herbs in China, have garnered attention for their dual dietary and therapeutic roles in NAFLD management (Li et al., 2021). Among these, Chenpi (Citri Reticulatae Pericarpium), derived from the dried mature peel of *Citrus reticulata* Blanco, and Rougui (Cinnamomi Cortex), obtained from the dried bark of *Cinnamomum cassia* Presl, are widely used in traditional medicine. Modern pharmacological studies demonstrated that Chenpi exhibited anti-inflammatory (Chen et al., 2021; Karthikeyan et al., 2021), antioxidant (Hijaz et al., 2020), hepatoprotective (Jiang et al., 2019), and antidiabetic potency (Ali et al., 2020). Rougui possessed antioxidant, anticholesterol (Hamidpour et al., 2015), antidiabetes, and anti-NAFLD properties (Hussain et al., 2021). Historically, the Chenpi-Rougui herb pair (CRP) was first recorded by Shiduo Chen in the *Stone chamber secret record* during the Qing dynasty, with functions of dispelling phlegm and dampness. Although pharmacological experiments have demonstrated the separate hepatoprotective effects of Chenpi and Rougui, the combined mechanism of this herb pair in treating NAFLD remains unclear.

Network pharmacology, rooted in interdisciplinary pharmacology and biology theories, constructs multi-compound, multi-target, multi-pathway interaction network, systematically assessing the intricate “ingredient-target-disease” relationships (Li and Zhang, 2013). However, its accuracy is constrained by database completeness and algorithmic biases (Zhang et al., 2019). Conversely, metabolomics is capable of mirroring the fluctuations of internal metabolites within biological systems in response to disease and pharmaceutical intervention (Nicholson and Lindon, 2008). It known for its comprehensiveness and capacity to capture dynamic processes, has emerged as a valuable tool for revealing the compatibility and intricate mechanisms underlying TCM (Liu et al., 2019; Ren et al., 2019). However, metabolomics primarily focused on the metabolic variation caused by external influences, overlooking endogenous mechanism of metabolite changes, upstream pathways and herb-targeted proteins (Qiu et al., 2023). Integrating these methods could bridge the gap between molecular targets and metabolic outcomes, thereby enhancing mechanistic understanding.

In this study, the chemical compositions of CRP decoction were characterized using UHPLC-QTOF-MS/MS. The synergetic hepatoprotective effects of CRP in high-fat diet (HFD) induced NAFLD mice were evaluated. Subsequently, network pharmacology was carried out to screen potential therapeutic targets based on the characterized chemical components. Serum metabolomic analysis was further performed to reveal potential metabolites and metabolic pathways involved in CRP and its monotherapy in the prevention and treatment of NAFLD. Finally, a joint analysis of targets from network pharmacology and serum metabolomic data, validated by RT-PCR experiments, was conducted to identify key targets. This study provided an integration strategy for uncovering the synergistic effects and compatibility mechanism of CRP against NAFLD, laying the foundation for the further development and utilization of this herb pair.

## 2 Materials and methods

### 2.1 Chemicals and reagents

Chenpi and Rougui were purchased from the Hubei Provincial Hospital of Traditional Chinese Medicine. The herbs were identified and authenticated by the taxonomist of the department of pharmacognosy (Hubei University of Chinese Medicine). Methanol (HPLC grade) was purchased from Fisher Scientific (Fair Lawn, NJ, United States). Deionized water was produced using a Milli-Q Ultra-pure water system (Millipore, Billerica, United States). Formic acid (HPLC grade, FA) was provided by Honeywell Company (USA). Simvastatin was obtained from Merck Pharmaceutical Co., Ltd. (Hangzhou, China). TC, HDL-C, and LDL-C assay kits were bought from Nanjing Jiancheng Bioengineering Co. Ltd. (Nanjing, China). RNA extraction solution, SweScript RT II First Strand cDNA Synthesis Kit (with gDNA remover) and Universal Blue SYBR Green qPCR Master Mix were bought from Wuhan Servicebio Technology Co., Ltd. (Wuhan, China).

### 2.2 Preparation of herbs extracts

A CRP decoction, composed of Chenpi (10 g) and Rougui (5 g), was made at a weight ratio of 2:1. Raw herb materials were cut into small pieces (approximately 1 cm × 1 cm) and soaked in distilled water (20 times volume of the herbs) for 0.5 h. Boiling the medicinal materials and water for 15 min produced the CRP decoction. After cooling to room temperature, the weight loss of the decoction was supplemented by adding pure water to maintain the concentration of CRP decoction at 5 g·L^−1^. Similar methods were used to prepare individual Chenpi and Rougui decoctions. All decoctions were prepared daily.

### 2.3 Analysis of chenpi, rougui and CRP extracts by UPLC-QTOF-MS/MS

In order to study the chemical constituents of CRP, the decoctions of Chenpi, Rougui and CRP were centrifuged by a 12,830 g high-speed centrifuge, and the supernatants after being filtered via a 0.22 μm microporous membrane were collected for analysis.

Waters ACQUITY UPLC system (Waters Corporation, Milford, MA, United States) was used for the Chromatographic. Separation was carried out on a Unitary C18 column (150 mm × 2.1 mm, 2.8 μm, Accrom Co., Ltd., Dilian, China). The elution system was 0.1% aqueous formic acid solution (A) and methanol (B), while linear gradient elution optimization was performed as follows: 0 min, 5% B; 20.0 min, 40% B; 40.0 min, 95% B; 45.0 min, 95% B; 46.0 min, 5% B; 50.0 min, 5% B. The flow rate was set at 0.3 mL/min, the column temperature was kept at 35°C, and the sample injection volume was 2.0 μL.

MS spectrometry experiment was performed using a Waters Xevo G2-XS QTOF and an electrospray ionization source (Waters, Mass., U.S.A.) in positive and negative electrospray ionization mode. Time-of-flight data were collected between m/z 100 to 1,200. The conditions for QTOF MS optimization were as follows: capillary voltage, 3000 V; cone voltage, 20 V; desolvation temperature, 500°C; source temperature, 100°C; desolvation gas flow, 600 L h^−1^. The collision energy spread was set at 30–40 eV. Besides, enkephalin was used to correct high-resolution molecular mass during acquisition to ensure accurate mass measurement.

### 2.4 Animal experiments

Male KM mice (n = 58, clean grade, weight = 20 ± 2 g) from Wuhan Institute of Biological Products Co., Ltd. (Wuhan, China) were used with certificate number: SCXK(e)2017-001. The mice were housed in stainless steel cages under standard environmental conditions (23°C ± 2°C, 40%–60% relative humidity, and 12 h light/12 h dark cycle) and allowed for free access to water and food. All animal experimental procedures were approved by the Ethical Committee of Hubei University of Traditional Chinese Medicine (approval number: 202203004).

After acclimation for 3 days, the mice were randomly divided into six groups: control group (n = 10), model group (n = 10), Simvastatin group (n = 8), CP (Chenpi) group (n = 10), RG (Rougui) group (n = 10) and CRP (Herb pair) group (n = 10). Mice, except for the control group, were fed with a high-fat diets (HFD) (Xu et al., 2023) contained 78.8% standard diet, 10% egg yolk, 10% lard, 1% cholesterol and 0.2% cholate for 5 weeks. During the administration of 28 days, the normal group and the model group had free access to drinking water. The Simvastatin group received a gavage dose of 40 mg. kg^−1^ of simvastatin. In order to simulate the traditional use method of the medicine food homology herbs, the CP group, RG group and CRP group were administered decoction (drinking freely) instead of drinking water.

After 5 weeks of feeding, the mice fasted for 12 h with water freely. Thereafter, the mice were anesthetized of 2% sodium pentobarbital (40 mg. kg^−1^) by intraperitoneal injection. Blood was collected from the orbit with ophthalmectomy, centrifuged at 2,680 g for 10 min to obtain serum samples, and stored at −80°C. Mice were euthanized for cervical dislocation, and liver samples were quickly separated from each mouse. Liver tissues were weighed, photographed, fixed in tissue fixative solution. The remaining liver tissues were frozen at −80°C until subsequent RT-PCR assays.

### 2.5 Biochemical analysis and histological evaluation

For biochemical analysis, the serum samples were thawed at 4°C before used. TC, LDL-C and HDL-C in serum were analyzed by enzyme calibration on 96-well plates. According to the manufacturer’s instructions, results were measured after setting the appropriate absorbance of the Microplate Reader. The sample values were then read off the standard curve, and the relative concentrations were calculated. For histological examination, the fixed liver tissues were embedded in paraffin, sectioned and stained with Oil Red O to observe the pathological changes under an optical microscope.

### 2.6 Network pharmacology analysis

Based on the 2D structures of the characterized compounds, the potential targets related CRP were predicted with probability >0.12 on SwissTargetPrediction (https://www.swisstargetprediction.ch/). NAFLD-related targets with a relevance score≥11were collected by searching from GeneCards (https://www.genecards.org/). String database (https://cn.string-db.org/) was used to build the protein-protein interaction (PPI) network. Visualization of PPI network and connections among herbs, components, targets and disease were performed using Cytoscape 3.9.0. software. The potential targets of CRP against NAFLD were analyzed using Metascape (https://metascape.org/) to decipher the biological mechanisms by Kyoto Encyclopedia of Genes and Genomes (KEGG) pathway enrichment and gene ontology (GO) functional enrichment.

### 2.7 Serum metabolomic analysis

#### 2.7.1 Analysis conditions of UPLC-QTOF-MS/MS

For LC-MS/MS analysis, each 40 μL of serum was mixed with 160 μL of acetonitrile (ACN) and 50 μL of internal standard (500 ng. mL^−1^ puerarin). The extraction was mixed vigorously for 5 min and centrifuged at 12,000 rpm for 10 min at 4°C to remove precipitated proteins. 200 μL of supernatant was taken into the sample vials waiting for LC-MS/MS analysis. To assess the reproducibility and reliability of the UPLC-QTOF-MS/MS system, quality control (QC) samples were prepared by mixing an equal volume of 58 serum samples and inserted at intervals of every six samples into the sequence.

Chromatographic separation was performed using a Waters ACQUITY UPLC system (Waters Corporation, Milford, MA, USA). Samples were injected into an ACQUITY UPLC BEH C18 column (100 × 2.1 mm, 1.7 μm) with a flow rate of 0.3 mL. min^−1^. The injection volume was 2.0 μL with the temperature staying at 35°C. Mobile phase A was water/formic acid (1,000: 1, v/v), and mobile phase B was methanol. The following binary gradient with linear interpolation was used: 0 min, 10% B; 15.0 min, 95% B; 20.0 min, 95% B; 21.0 min, 10% B; 25.0 min, 10% B. Other conditions of mass spectrometry were the same as “2.3”part, except for cone voltage is 60 V.

#### 2.7.2 Data processing and analysis

Mass Lynx 4.1 (Waters, MA, United States) was used to process the raw data from UPLC-QTOF-MS/MS. The acquired UPLC-MS/MS data were imported to MarkerLynx XS (Waters, MA, United States) to perform noise filtering, peak extraction and peak alignment, with the following parameters: noise elimination level (40), peaks’ retention time mass tolerance (0.01 Da). A preliminary data matrix including m/z, retention time, and relative peak intensity was used for multivariate statistical analysis. Then, filter the data matrix with eliminate missing value exceeded 60%, and log2 transformation were used to transform the data to a normal distribution.

The processed data matrix was imported into SIMCA-P 14.1 (Umetrics, Umeå, Sweden) for multivariate statistical analysis. The analyses of principal component analysis (PCA) and orthogonal partial least squares discriminant analysis (OPLS-DA) were conducted to visualize the metabolic variations among experimental groups. In addition, a 999-time permutation test was conducted to examine model for overfitting. Variables elected as potential biomarkers with the satisfied criteria VIP (variable importance in the projection) > 1.5, *p* < 0.05. Differential metabolites were searched and identified (mass difference <10 ppm) with exact mass, retention time and MS/MS fragmentation by searching the human metabolite database (HMDB) (http://www.hmdb.ca) and METLIN database (http://metlin.scripps.edu). After decoction administration, the differential metabolites with fold change (FC) > 1.1 or FC < 0.9 were considered to have regulatory effects. After z-score standardization of the content of metabolites by SPSS 13.0 software, a visual heat map was then performed to reflect changes in differential metabolites. Ultimately, the Kyoto Encyclopedia of Genes and Genomes database (KEGG) (http://genome.jp/kegg/) were used to confirm the related pathways.

### 2.8 Integrated network pharmacology and metabolomics analysis

The targets of differential metabolites were inquired in the STITCH (http://stitch.embl.de/). The overlapping targets between the differential metabolite related targets and potential therapeutic targets were considered potential targets for the treatment of NAFLD with CRP. The interactions between potential active compounds, target proteins, and metabolic pathways were mapped by Cytoscape 3.9.0. On this basis, RT-PCR analysis was used to further verify these potential targets.

### 2.9 Real-time polymerase chain reaction analysis

Liver samples were lysed using Trizol reagent (Servicebio, Wuhan, China) to obtain the total RNA. Nanodrop 2000 (Thermo Fisher Scientific) was used to measure the purity and concentration of RNA. The reverse transcription kit (Servicebio, Wuhan, China) was applied to reverse transcribe RNA into cDNA. The CFX96 Real-Time polymerase chain reaction System (Bio-Rad) was performed to detect the mRNA expression levels using SYBR Green qPCR Master Mix kit (Servicebio, Wuhan, China). The relative expression of mRNA was normalized to Glyceraldehyde-3-phosphate dehydrogenase (GAPDH) and analyzed using the 2^−ΔΔCT^ method. The primer sequences used for PCR were shown in Supplementary Table S1.

### 2.10 Statistical analysis

Data were expressed as mean ± standard deviation (SD). Statistical analysis was performed by unpaired t-test using GraphPad Prism 8.0. A *p*-value of <0.05 was considered to be statistically significant.

## 3 Results

### 3.1 Characterization of components in CRP

**FIGURE 1:**
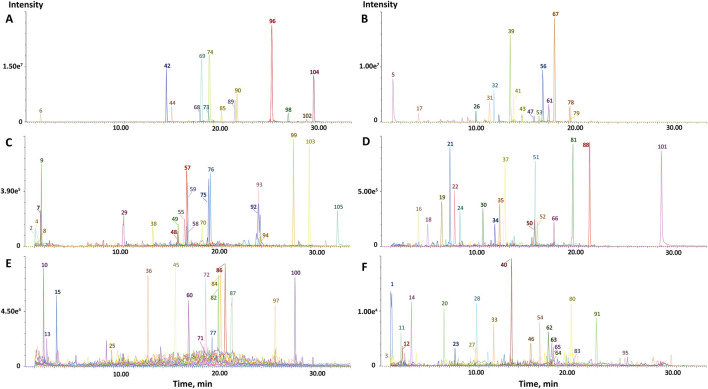
Extract ions chromatography (EIC) of CRP based on UPLC-QTOF-MS/MS. **(A)** In the positive ion mode, the peak intensity is 7.80e^5^ ∼ 3.00e^7^; **(B)** In the negative ion mode, the peak intensity is 1.00e^6^∼2.00e^7^; **(C)** In the positive ion mode, the peak intensity is 9.00e^4^∼7.80e^5^; **(D)** In the negative ion mode, the peak intensity is 2.00e^4^∼1.00e^6^; **(E)** In the positive ion mode, the peak intensity is 0.00∼9.00e^4^; **(F)** In the negative ion mode, the peak intensity is 0.00∼2.00e^4^.

The characterization of components in CRP was conducted using UPLC-QTOF-MS/MS analysis. Based on ingredient databases summarized from literature reports, a total of 105 constituents were characterized from the extract of CRP in this study. The extracted ion chromatograms of all compounds were shown in Figure 1. The name, category and mass spectrometry information of all compounds were shown in Supplementary Table S2. Among the characterized compounds, 53 compounds were identified in CP and 52 compounds in RG. Notably, flavonoids constituted the largest share, accounting for 37.14% of the total constituents. Additionally, 20% of the compounds were classified as phenolic acids, underscoring the diverse array of bioactive compounds present in CRP.

### 3.2 Hepatoprotective activity of CRP in NAFLD

In this study, all mice were in good health during the experiment at a given dose of administration. Decoction intakes were monitored daily, and no significant differences were observed between groups. The physical and biochemical analyses of each group were shown in Table 1. Compared to control group, 4 weeks of HFD feeding resulted in significantly increased the weight, serum TC and LDL-C levels. Although there was no significant difference in liver index and HDL-C level of NAFLD mice, some changes were observed compared to control group. The simvastatin group only exhibited significant reductions in the weight and TC level compared to the model mice. Treatment with CP, RG, and CRP lowered the elevated body weight by 14.21%, 6.55% and 6.37%, and decreased the liver index by12.83%, 25.87% and 21.18%, respectively, compared to the model group. All treatment groups (CP, RG, and CRP) showed significant decrease in TC and LDL-C levels, with CP showing the highest reduction in TC (26.69%) and RG showing the highest reduction in LDL-C (65.25%). As for the serum HDL-C level, the treatment groups demonstrated a significant increase compared to the model group, with CP showing the highest increase of 33.51%, followed by RG (27.75%) and CRP (19.37%).

**TABLE 1:** Therapeutic and prophylactic impacts of CRP and single drug on NAFLD mice (mean ± S.D.).

Group	Weight	Liver index (%)	TC	HDL-C	LDL-C
control	47.77 ± 4.01	4.30 ± 0.51	4.96 ± 0.34	4.72 ± 0.32	0.19 ± 0.06
model	54.18 ± 5.50^**^	4.91 ± 1.11	7.12 ± 0.77^***^	3.82 ± 1.15	1.18 ± 0.38^***^
Simvastatin	49.40 ± 5.44^#^	4.67 ± 0.56	5.27 ± 0.66^##^	3.77 ± 0.60	0.94 ± 0.31
CP	46.48 ± 3.16^##^	4.28 ± 0.48	5.22 ± 0.90^###^	5.10 ± 0.75^#^	0.49 ± 0.12^###^
RG	50.63 ± 5.28	3.64 ± 0.39^##^	5.37 ± 0.64^##^	4.88 ± 0.48^#^	0.41 ± 0.08^###^
CRP	50.73 ± 3.37^#^	3.87 ± 0.60^##^	6.36 ± 0.61^#^	4.56 ± 0.56^#^	0.50 ± 0.13^###^

TC, total cholesterol; LDL-C, low-density lipoprotein cholesterol; HDL-C, high-density lipoprotein cholesterol; Liver index (%) = liver weight/body weight × 100%.

**p < 0.01, *** *p* < 0.001 significantly different from the control group.

# p < 0.05, ## *p* < 0.01, ###*p* < 0.001 significantly different from the model group.

**FIGURE 2:**
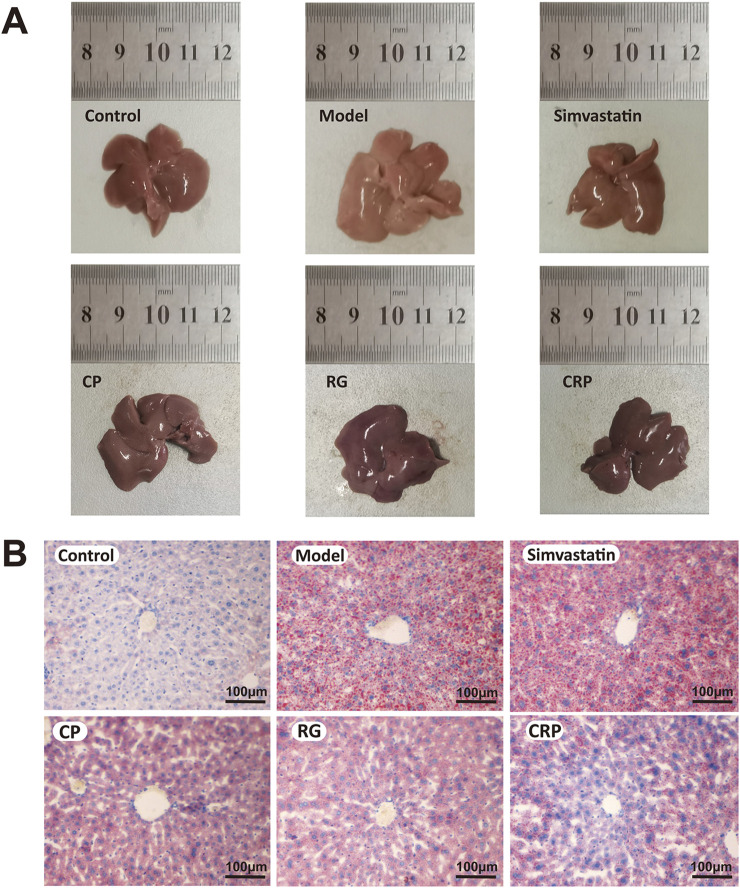
Liver histopathological examination. **(A)** Appearance picture of liver taken at the same height, location, and resolution. **(B)** Oil red O staining showed the distribution of lipid droplets in the liver of each group (×100). A bar = 100 μm.

As shown in Figure 2, liver morphological and histological examinations revealed distinct differences between the control and treatment groups. The liver of the control group appeared ruddy and shiny, with no significant lipid accumulation and steatosis in the hepatocytes. By contrast, the liver of the model group displayed white and swollen appearance with extensive lipid droplets. The simvastatin group showed some improvement, while the CP, RG and CRP groups displayed notable improvement in liver morphology and reduction in hepatocyte lipids. The CRP group, in particular, demonstrated liver morphology and histology closer to normal.

In conclusion, CP exhibited better effects in preventing weight gain and blood lipid increase, while RG was more conducive to preventing and treating liver swelling. CRP demonstrated extensive effects in preventing weight gain, serum cholesterol increases and liver swelling.

### 3.3 Network pharmacology analysis

A total of 105 compounds from CRP were searched within the Swiss Target Prediction database, leading to the prediction of 279 probable targets (Supplementary Table S3). Simultaneously, 864 targets associated with NAFLD were obtained in GeneCards according to the relevance score (Supplementary Figure S1). By matching the compound-related targets with the targets of NAFLD, 31 components and 70 potential targets against NAFLD for CRP were identified, suggesting that these targets might contribute to the hepatoprotection of CRP. To elucidate the interactions among these targets, cytoscape was conducted to construct herb-component-target-disease interaction network, as illustrated in Supplementary Figure S2. This network elucidated the multifaceted interactions facilitating CRP’s potential therapeutic effects on NAFLD. Furthermore, compounds with higher degree values in this network were mainly flavonoids, limonins, phenolic acids and diterpenoids, indicating their potential roles as the principal bioactive constituents in the herbal combination for alleviating NAFLD.

**FIGURE 3:**
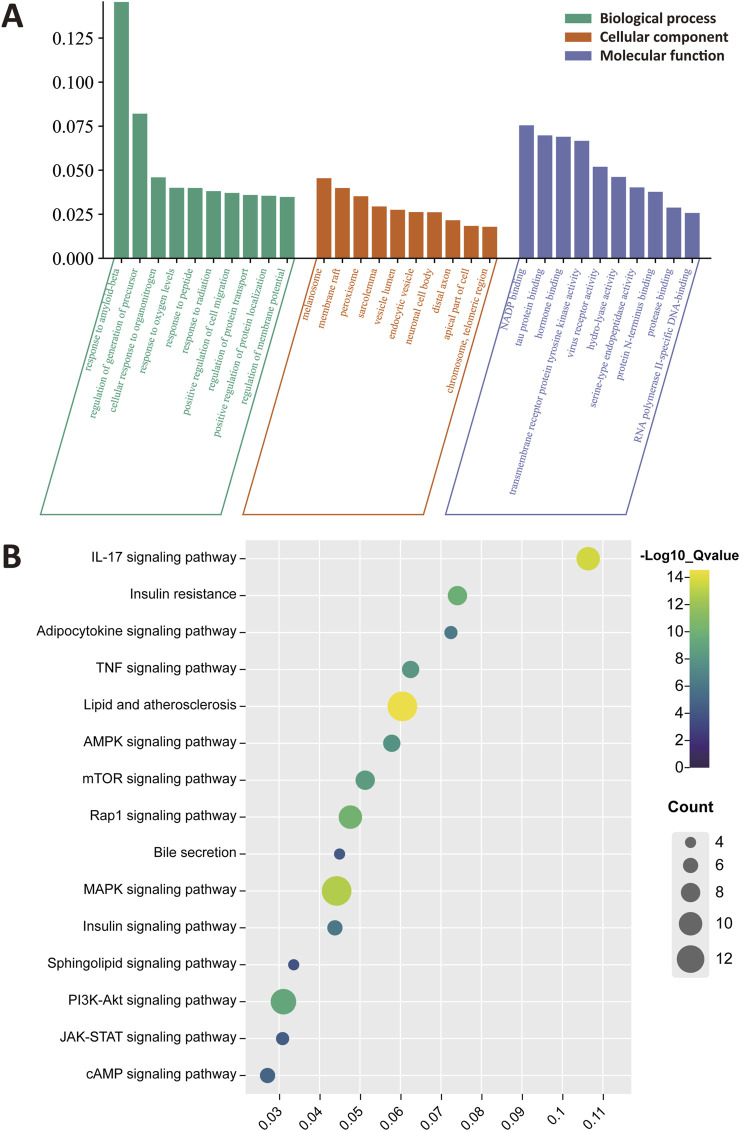
Functional enrichment and pathway analysis of CRP. **(A)** Gene ontology (GO) enrichment analysis. The top 10 enrichment terms were identified for each functional category. **(B)** Kyoto encyclopedia of genes and genomes (KEGG) enrichment analysis. All pathways have a p-value <0.05.

To clarify the biological roles of CRP for treating NAFLD, the GO functions and KEGG pathway analysis were performed. As shown in Figure 3A, the results of enrichment were sequenced with gene ratio, and the top 10 analysis results for cellular component (CC), biological process (BP), and molecular function (MF) were mapped. These pathways primarily encompassed response to amyloid-beta, regulation of generation of precursor, melanosome, membrane raft and NADP banding. The top 10 analysis results of KEGG enrichment were shown in Figure 3B, emphasizing pathways such as the IL-17 signaling pathway, insulin resistance, Rap1 and MAPK signaling pathway.

### 3.4 Untargeted metabolomics analysis

**FIGURE 4:**
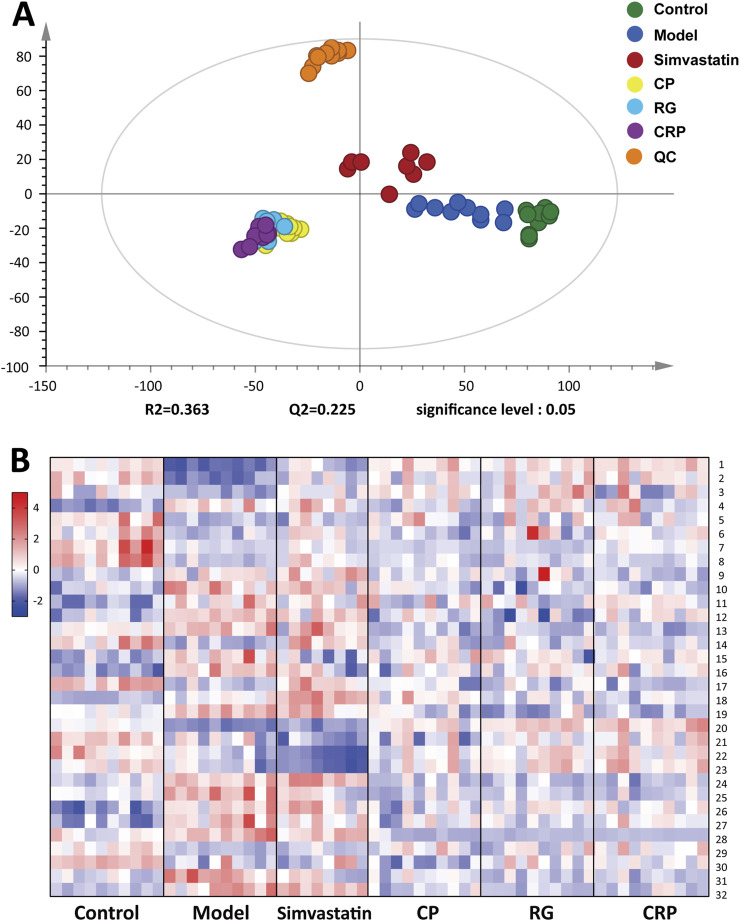
**(A)** PCA score plot of Control, Model, Simvastatin, CP, RG, CRP and QC groups (*R* ^2^ = 0.363, Q^2^ = 0.225). **(B)** Heatmap of differential metabolites relative abundance levels among the groups. The color from blue to red indicated the content from low to high.

According to the total ion chromatogram (TIC) of QC samples, the retention time and peak intensity of each peak showed good repeatability (Supplementary Figure S3). Furthermore, the scoring plot of PCA showed that QC samples, represented by the orange dots, were aggregated tightly in an unsupervised mode (Figure 4A), confirming the high repeatability of the analytical methods and instrumentation, thus ensuring data suitability for further analysis. As shown in Figure 4A, an obvious separation between the control and model group was observed in the PCA scoring plot, indicating that the mice serum metabolic profiles changed after HFD feeding. Meanwhile, the treatment groups were separated from the model group respectively, with partial overlap between the CP, RG and CRP groups, suggesting that the herb treatment groups (CP, RG and CRP) altered the serum metabolic profile of NAFLD mice and might exert similar intervention effects. Subsequently, a supervised OPLS-DA model was fitted to determine the metabolic differences between the control, model and administration group. As shown in Supplementary Figure S4A–E, R^2^Y and Q^2^ showed good fitting ability and predictive ability of the OPLS-DA models. Meanwhile, 999-time permutation tests were conducted to evaluate the reliability of the OPLS-DA models (Supplementary Figure S4F–J). The separation of observations between two groups occurred in the horizontal direction (x-axis). The vertical (y-axis) direction expressed within class variability. All Q^2^-values on the left were lower than the original points on the right and the blue regression line of the Q^2^-points intersected the vertical axis below zero, indicating the OPLS-DA models were not over-fitting and had well prediction ability.

Based on the OPLS-DA model of the control and model group, the differential metabolites with the predetermined rules (VIP > 1.5 and *p* < 0.05) were screened. As a result, a total of 139 differential metabolites were identified as endogenous biomarkers involved in pathogenesis of NAFLD. Following, the differential impact of CP, RG, and CRP on these metabolites was evaluated through fold change analysis (FC > 1.1 or <0.9). Finally, 32 specific metabolites exhibited callback trends in herb treatment groups compared to the model group, and their identification information were listed in Supplementary Table S4. The corresponding information, changing trend and fold change between groups were shown in Table 2.

**TABLE 2:** The differential metabolites characterized in plasma and their change trends after treatment with different herbs.

No	RT (min)	Metabolites	VIP^a^	Trend^b^				Fold change			
				M/C	CP/M	RG/M	CRP/M	M/C	CP/M	RG/M	CRP/M
1	16.91	Ubiquinone-2	2.27	↓^***^	↑^***^	↑^***^	↑^***^	0.4293	2.4621	2.4185	2.6013
2	16.40	n6-[2-(4-Aminophenyl)ethyl] adenosine	2.28	↓^***^	↑^***^	↑^***^	↑^***^	0.3033	2.5222	2.7869	2.9110
3	16.41	Dodec-7-enedioylcarnitine	2.45	↓^**^	↑^***^	↑^***^	↑^**^	0.4694	2.6957	3.0000	1.9130
4	16.19	Stearoylcarnitine	1.99	↑^***^	↓^**^	—	↓	1.7716	0.7640	0.9468	0.8903
5	15.23	Hydroxyeicosatetraenic acid	2.38	↓^***^	↑^*^	↑	↑^**^	0.5169	1.4853	1.4089	1.4896
6	14.74	Hydroxylinolenic acid	2.26	↓^**^	—	↑	↑^**^	0.6562	1.0171	1.4007	1.3082
7	16.01	Octadecatetraenoic acid	2.09	↓^***^	—	—	↑^*^	0.2476	0.7725	0.6497	1.4341
8	14.60	Cosahexaenoic acid	2.21	↓^***^	↑	↑	↑	0.2971	1.1756	1.1945	1.1148
9	15.31	Sphinganine	2.16	↑^*^	↓^*^	—	↓^**^	1.6676	0.7383	0.9500	0.5328
10	17.47	Cer(t18:0/16:0)	1.67	↑^**^	↓^**^	↓^**^	↓^**^	1.5277	0.7234	0.6137	0.6698
11	16.23	Ganglioside GA2 (d18:1/9Z-18:1)	1.98	↑^***^	↓	↓	↓	3.3730	0.7555	0.7122	0.8617
12	16.46	Ganglioside GM3 (d18:0/12:0)	2.17	↑^***^	↓^**^	↓^**^	↓^**^	1.8587	0.6438	0.4441	0.7645
13	18.09	DG(PGD1/i-15:0)	1.55	↑^**^	↓^***^	↓^***^	↓^***^	1.4457	0.3979	0.4442	0.4129
14	16.36	PS(20:4/18:0-2OH)	2.23	↓^**^	↑	↑	↑^*^	0.4219	1.2923	1.4076	1.3246
15	16.48	PC(LTE4/22:0)	1.94	↑^**^	↓	↓	↓	2.331	0.8368	0.7244	0.7380
16	16.84	PE(20:4-2OH/DiMe)	2.16	↑^***^	↓^**^	↓^**^	↓^*^	1.5188	0.8058	0.8716	0.8627
17	16.48	PG(LTE4/20:2)	2.29	↓^***^	↑	↑	↑	0.3269	1.2266	1.1758	0.8945
18	16.81	PG(i-14:0/i-13:0)	1.51	↑^***^	—	↓	—	1.3426	1.1064	0.8314	0.8861
19	17.65	LysoPI(20:4)	1.80	↑^***^	↓^***^	↓^**^	↓^***^	1.4862	0.5981	0.4753	0.4847
20	15.88	LysoPE(22:6)	1.86	↓^***^	↓^***^	↓^***^	↓^***^	0.7200	3.7540	4.5755	4.9711
21	16.16	LysoPE(22:5)	1.74	↓^*^	—	—	↑	0.8580	0.9277	1.0461	1.1837
22	17.06	LysoPE(18:0)	1.87	↓^**^	↑	↑^**^	↑^**^	0.8919	1.1876	1.2895	1.3398
23	16.33	LysoPE(16:0)	2.06	↓^**^	↑	↑^**^	↑^**^	0.7640	1.2106	1.3439	1.4187
24	16.88	LysoPC(P-18:0)	2.03	↑^***^	↓^**^	↓^***^	↓^***^	1.5161	0.638	0.5911	0.6071
25	17.45	LysoPC(O-18:0)	1.92	↑^***^	↓^***^	↓^***^	↓^***^	1.6752	0.5198	0.5235	0.5200
26	16.46	LysoPC(18:1)	2.02	↑^***^	↓^*^	—	↓^*^	1.3761	0.8521	0.9246	0.9020
27	16.68	LysoPC(20:2)	2.08	↑^***^	↓^**^	↓^**^	↓*	2.0623	0.6861	0.6509	0.7576
28	15.99	Dihydrotestosterone diglucuronide	1.54	↑^**^	↓^***^	↓^***^	↓^***^	2.1572	0.1209	0.1119	0.1148
29	18.59	PGD2 ethanolamide	2.12	↓^***^	↑^*^	↑	↑^**^	0.4282	1.9396	1.7991	2.7251
30	16.66	LTB4 ethanolamide	2.43	↓^**^	—	↑	↑	0.5078	0.6448	1.1931	0.6564
31	16.81	Arachidonic acid	1.64	↑^***^	↓^**^	↓^***^	↓^***^	1.8160	0.5933	0.5165	0.5141
32	15.31	Sulfolithocholylglycine	1.94	↑^***^	↓^**^	↓^***^	↓^**^	2.0117	0.5717	0.5070	0.5558

The changes of differential metabolites were labelled with (↓) downregulated (Fold change <0.9) and (↑) upregulated (Fold change >1.1).

^a^ Calculated by the OPLS-DA, model based on metabolites of control and model group.

^b^ The differential metabolites were not regulated in the administration groups compared with the model group.

M/C means the model group compared with the control group; CP/M, RG/M and CRP/M means CP, RG, and CRP, groups compared with the model group, respectively.

* *p* < 0.05, ** *p* < 0.01, *** *p* < 0.001 significantly different from the model group.

Besides, a heatmap was produced to visually depict the distribution of 32 potential metabolites among groups (Figure 4B). Compared to the control group, the model group exhibited significant alterations in metabolites, with 15 demonstrating marked decreases and 17 displaying significant increases (*p* < 0.05). The herbal treatment groups regulated a subset of these metabolites, with each herb exerting different regulatory effects. CP regulated 27 metabolites, with 20 exhibiting substantial differences. RG modulated 27 metabolites, among which 18 displayed significant differences. Notably, RG uniquely regulated one metabolite, PG (i-14:0/i-13:0). CRP regulated 31 metabolites, with 24 exhibiting noteworthy differences, and uniquely regulated 2 metabolites, Octadecatetraenoic acid and LysoPE (22:5). CRP displayed the most comprehensive regulatory effect on altered metabolites compared to the two individual herbs.

**FIGURE 5:**
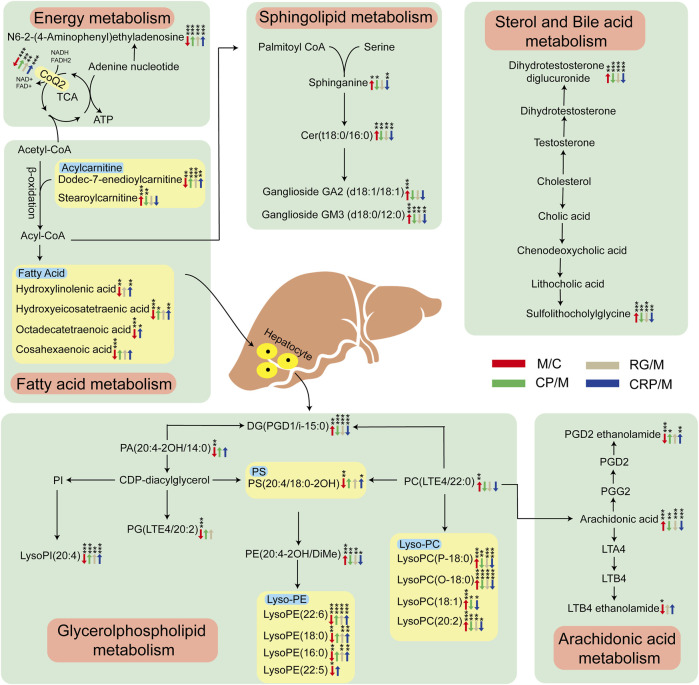
Metabolic disorders and regulatory network diagram. The variation trend of metabolites in model group compared with control group is indicated as red arrows, while green, gray and blue arrows represent the regulatory trend in CP, RG and CRP group relative to model group, respectively. *p < 0.05, **p < 0.01, ***p < 0.001 significant difference level between corresponding two groups.

To explore the potential links between these callback metabolites, a metabolic network affected by HFD-induced NAFLD and regulated by the administration were depicted in Figure 5. Six pivotal metabolic pathways were identified as responsible for the hepatoprotective effects of CP, RG and CRP: energy metabolism, fatty acid metabolism, glycerophospholipid metabolism, sphingolipids metabolism, arachidonic acid metabolism, sterol and bile acid metabolism. Both CP and RG exhibited significant regulation in energy metabolism and sterol and bile acid metabolism. However, CP demonstrated more pronounced effects in sphingolipid metabolism, particularly in sphinganine regulation. Conversely, RG exhibited greater regulation of metabolites in fatty acid metabolism and arachidonic acid metabolism. Furthermore, in glycerophospholipid metabolism, CP prominently affected lysophosphatidylcholines (Lyso-PC), while RG notably influenced lysophosphatidylethanolamines (Lyso-PE). In contrast, CRP demonstrated a broader regulatory spectrum, impacting multiple metabolites across these six pathways.

### 3.5 Integrated analysis

**FIGURE 6:**
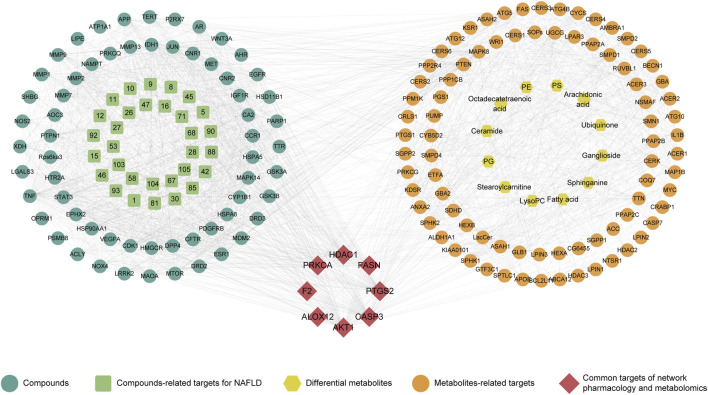
Combination of network pharmacology and serum metabolomics.

For an in-depth understanding of the compatibility mechanism for CRP, an interaction network was conducted according to network pharmacology and metabolomics. In this process, 89 differential metabolites-related targets involving discriminant metabolites were obtained (Supplementary Figure S5). The integrated analysis highlighted 8 key targets, including ALOX12 (arachidonate 12-lipoxygenase), FASN (fatty acid synthase), AKT1 (thymoma viral proto-oncogene 1), CASP3 (caspase 3), F2 (coagulation factor II), HDAC1 (histone deacetylase 1), PTGS2 (prostaglandin-endoperoxide synthase 2), PRKCA (protein kinase C). These targets were found to be relevant in the modulation of NAFLD by CRP, as depicted in Figure 6.

### 3.6 Validation of the key targets

**FIGURE 7:**
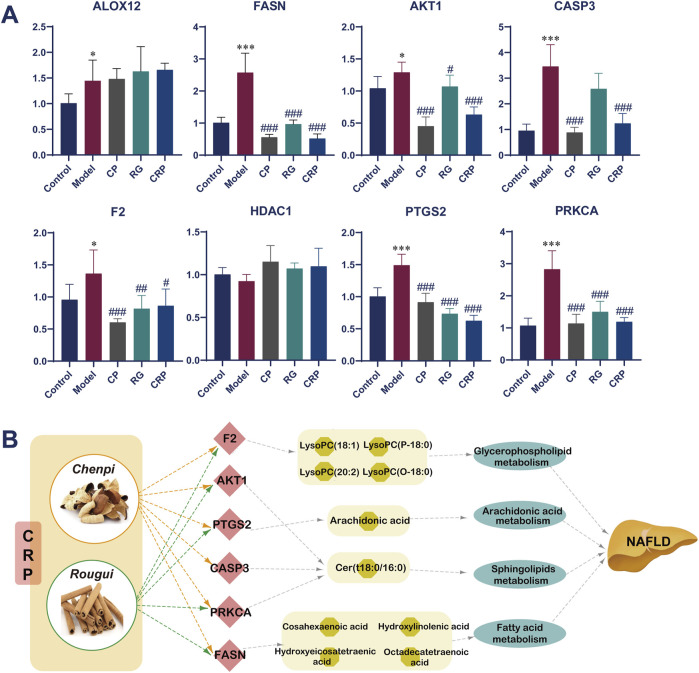
**(A)** Effect of CRP on the mRNA expression of targets in the prevention of NAFLD detected by RT-PCR (n = 6). Mann-Whitney U test was used to calculate significant difference. ^*^ *P* < 0.05, ^***^ *P* < 0.001, compared with control group. ^#^ *P* < 0.05, ^##^ *P* < 0.01, ^###^ *P* < 0.001, compared with model group. Abbreviations: ALOX12: arachidonate 12-lipoxygenase; FASN: fatty acid synthase; AKT1: thymoma viral proto-oncogene 1; CASP3: caspase 3; F2: coagulation factor II; HDAC1: histone deacetylase 1; PTGS2: prostaglandin-endoperoxide synthase 2; PRKCA: protein kinase C. **(B)** Involved pathways and metabolisms of validated targets.

In order to validate the robustness of our findings obtained from metabolomics analysis and network pharmacology, real-time polymerase chain reaction (RT-PCR) experiments were conducted to assess the expression of 8 targets. As shown in Figure 7A, there was a substantial elevation in the expression levels of ALOX12, FASN, AKT1, CASP3, F2, PTGS2 and PRKCA in the model group compared to control group. Following treatment with CP, RG, and CRP, there was a significant reduction in the mRNA levels of FASN, AKT1, CASP3, F2, PTGS2, and PRKCA compared to the model group. However, it was worth noting that CP, RG and CRP had no significant regulatory effect on the expression of ALOX12 and HDAC1 in NAFLD mice. Finally, 6 key targets, namely, FASN, AKT1, CASP3, F2, PTGS2 and PRKCA, were identified based on these findings.

Furthermore, a H-T-M-P-D (Herb-Target-Metabolite-Pathway-Disease) network analysis of the synergistic mechanism of CRP for preventing and treating NAFLD was shown in Figure 7B. This network elucidated how the modulation of these 6 key targets could potentially alter 10 specific metabolites, thereby influencing pathways associated with the glycerophospholipid, fatty acid, arachidonic acid, and sphingolipid metabolism.

## 4 Discussion

This study systematically compared the preventive effects of CRP on NAFLD with its two individual herbs, Chenpi and Rougui, and explored the pharmacological mechanisms of CRP using an integrated approach of untargeted metabolomics and network pharmacology. The results from animal experiments demonstrated that CRP more effectively inhibited the progression of NAFLD compared to either herb alone. This enhanced efficacy can be attributed in part to the synergistic effects of Chenpi and Rougui in CRP. Metabolomic analysis revealed that CRP regulated a broader range of metabolites, with two metabolites, Octadecatetraenoic acid and LysoPE (22:5), uniquely influenced by the combination of Chenpi and Rougui. In contrast, each of the individual herbs regulated 27 metabolites, while CRP regulated 31, further highlighting its broader regulatory scope. For example, in glycerophospholipid metabolism, Chenpi prominently affected LysoPC, and Rougui notably influenced LysoPE. CRP, however, combined these effects, demonstrating an additive influence on this pathway. This combination not only expands the spectrum of regulated metabolites but also potentially enhances the overall modulation of glycerophospholipid metabolism, which is crucial in NAFLD pathogenesis. Network pharmacology further confirmed that CRP had a more extensive impact on key metabolic pathways and protein targets, validated by RT-PCR, including FASN, AKT1, CASP3, F2, PTGS2, and PRKCA. These proteins are pivotal in mediating CRP’s beneficial effects on NAFLD.

The combination of Chenpi and Rougui in CRP leads to synergistic effects on four key metabolic pathways: glycerophospholipid, fatty acid, arachidonic acid, and sphingolipid metabolism. These effects contribute to CRP’s comprehensive modulation of NAFLD. Glycerophospholipids, particularly LysoPC and LysoPE, were identified as prominent differential metabolites in NAFLD mice. LysoPC, a pro-inflammatory glycerophospholipid, is implicated in the pathogenesis of NAFLD due to its ability to promote hepatic inflammation and fibrosis. Elevated LysoPC levels can lead to increased oxidative stress and disruption of cellular membranes, thereby exacerbating liver injury (Han et al., 2008). Factor II (F2) is a key protein in the blood coagulation cascade. It is converted into thrombin, which then facilitates the conversion of fibrinogen to fibrin, leading to clot formation. In conditions like NAFLD, altered coagulation and increased expression of procoagulant factors, including F2, can contribute to liver inflammation and fibrosis (Lallukka et al., 2017). Our findings suggested that CRP modulated the coagulation pathway via F2, leading to a reduction in thrombin levels. This modulation likely resulted in decreased platelet activation and inflammatory response, thereby reducing LysoPC levels in the liver. By attenuating LysoPCs (18:1, 20:2, P-18:0, O-18:0)-induced inflammation, CRP helped stabilize hepatocyte membranes, mitigate liver injury, and slow NAFLD progression.

Ceramide (Cer), a core position of the sphingolipids metabolism network, can cause intracellular mitochondrial depolarization, leading to inflammation and oxidative stress in hepatocytes (Mari and Fernandez-Checa, 2007). And sphingolipid metabolites (such as ceramide, ganglioside GM3, etc.) can inhibit insulin signaling and action (Holland et al., 2007). Our study indicated that CRP influences ceramide metabolism through the modulation of key signaling pathways involving AKT1, CASP3, and PRKCA. AKT1 plays a key role in cell growth, such as glucose metabolism, apoptosis, cell proliferation, transcription and cell migration (Franke, 2008). Diet intervention could increase the lipid oxidation capacity of liver and improve NAFLD by regulating AKT and related targets (Gao et al., 2020). CRP downregulated AKT1, which may reduce the activation of downstream signaling pathways involved in lipid synthesis and cell survival. This downregulation led to decreased ceramide synthesis, improving insulin sensitivity and reducing hepatic lipid accumulation. The inhibition of AKT1 may also prevent excessive cell proliferation and contribute to reducing hepatic inflammation (Ding et al., 2023). CASP3 is a critical executor of apoptosis in hepatocytes. The formation of apoptotic bodies activates CASP3, leading to DNA degradation and cell apoptosis (Decker and Muller, 2002). CRP’s ability to inhibit CASP3 prevented the activation of apoptotic pathways triggered by ceramide accumulation, thus protecting hepatocytes from apoptosis and preserving liver function. In the animal model of NAFLD, PRKCA was activated and expressed at elevated levels and played a role in endoplasmic reticulum stress signaling and cell death (Greene et al., 2010). By regulating PRKCA, CRP reduced ceramide-induced endoplasmic reticulum stress, thereby attenuating hepatic inflammation and cell death.

Arachidonic acid (AA) is a pro-inflammatory lipid whose metabolism contributes to the production of eicosanoids, which are potent mediators of inflammation in NAFLD (Sonnweber et al., 2018). PTGS2 is a key enzyme in the conversion of AA to inflammatory prostaglandins such as PGE2 and PGD2 (Chen et al., 2019). CRP was found to downregulate PTGS2, which led to a reduction in pro-inflammatory prostaglandins, thereby decreasing oxidative stress and inflammatory signaling in the liver. By modulating AA metabolism, CRP mitigated the inflammation-driven progression of NAFLD, promoting hepatic recovery and function.

Polyunsaturated fatty acids (PUFAs) such as eicosatetraenoic acid, linolenic acid, and octadecatetraenoic acid are essential for maintaining hepatic health due to their anti-inflammatory and hepatoprotective properties (Rich, 1993; Hanna and Hafez, 2018). Fatty acid synthase, encoded by FASN, is an enzyme involved in the synthesis of long-chain fatty acids. Recent clinical trials of FASN inhibitors TVB-2640 and FT-4101 had confirmed the therapeutic potential of FASN targeted therapy for NAFLD (Hu et al., 2021). CRP’s influence on fatty acid metabolism was mediated through the regulation of FASN. By downregulating FASN, CRP reduced the synthesis of saturated fatty acids, which were substrates for the production of lipotoxic metabolites like ceramide and diacylglycerol. This modulation enhanced the synthesis of beneficial PUFAs, thereby decreasing hepatic steatosis and inflammation. The increase in PUFA levels following CRP treatment supported their protective roles against liver damage and inflammation, suggesting that CRP improved lipid metabolism and restores hepatic function in NAFLD. The enhanced production of PUFAs may also contribute to the reduction of lipid peroxidation and oxidative stress in the liver.

Despite these findings, several limitations warranted consideration. Firstly, while RT-PCR confirmed mRNA expression changes of key targets, protein-level validation was absent, and the mechanistic roles of these proteins in CRP’s effects remained speculative. To better understand the impact of CRP on hepatic lipid uptake and oxidation, further investigations on crucial upstream regulators of lipid metabolism, such as CD36 (Glatz et al., 2022), AMP-activated protein kinase (AMPK) (Zhang et al., 2024), peroxisome proliferator-activated receptors (PPARs) (Montaigne et al., 2021), or forkhead box O1 (FoxO1) (Kim et al., 2016), were essential to confirm their functional contributions. Secondly, metabolite identification relied on database matching without standard reference confirmation, which may introduce annotation uncertainties. Thirdly, network pharmacology predictions were constrained by database completeness and algorithmic biases, potentially overlooking novel targets or pathways. These limitations highlighted the need for experimental validation and expanded multi-omics integration in future research. Such efforts will be crucial for a more indepth understanding of the mechanisms of CRP in treating NAFLD and for the further development of CRP as a therapeutic agent.

## 5 Conclusion

In this study, the integrative analysis of metabolomics and network pharmacology data revealed that CRP exerted its therapeutic effects in NAFLD through a multi-targeted approach. CRP exhibited superior efficacy and a wider range of metabolic regulatory effects in providing protection against NAFLD in comparison to the individual herbs. By modulating key metabolic pathways and gene expressions, CRP effectively reduced inflammation, improved lipid metabolism, and enhanced overall hepatic health. These findings underscore CRP’s potential as a promising therapeutic agent against NAFLD, offering a multifaceted strategy to counteract the disease’s progression.

## Data Availability

The original contributions presented in the study are included in the article/Supplementary Material, further inquiries can be directed to the corresponding authors.
