# Supplementary material for: Network pharmacology combined with untargeted metabolomics reveals the intervention mechanism and compatibility of chenpi-rougui herb pair in nonalcoholic fatty liver disease
Source: Front Mol Biosci. 2025 Mar 13;12:1553162. doi: 10.3389/fmolb.2025.1553162 (PMC11966411; doi:10.3389/fmolb.2025.1553162)
Supplement: Supplementary file 1 [file DataSheet1.docx]

Supplementary data

**Network pharmacology combined with untargeted** **metabolomics reveals the intervention mechanism and** **compatibility of** **Chenpi-Rougui herb pair in nonalcoholic fatty liver disease**


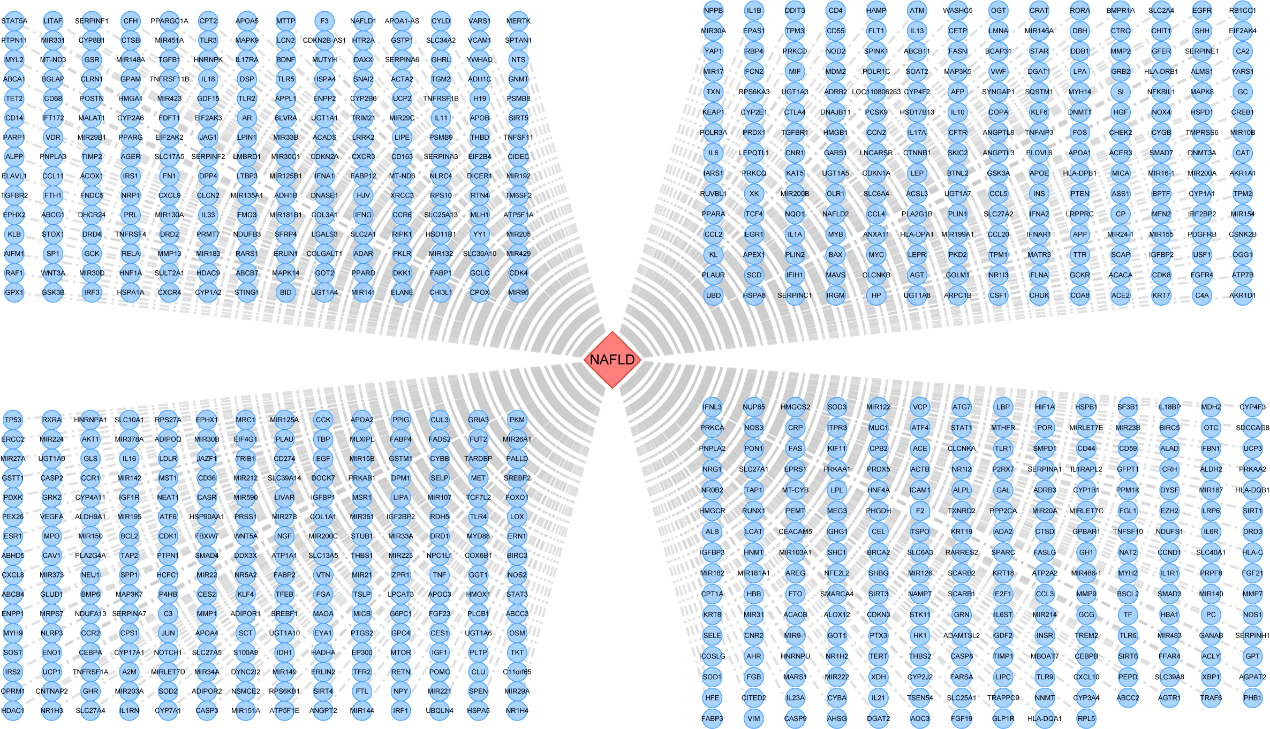


Figure S1. Screened targets related to nonalcoholic fatty liver disease.


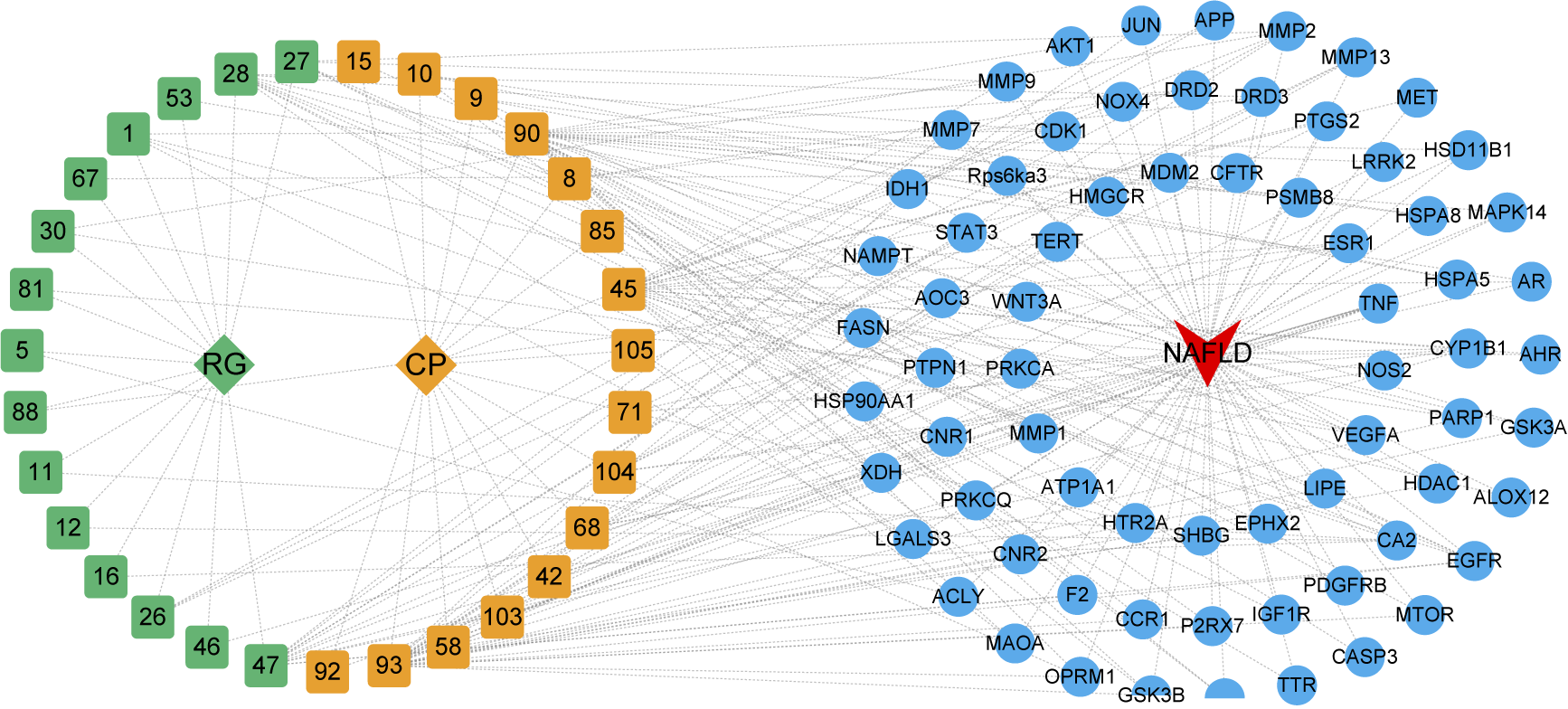


Figure S2. Herb-component-target-disease interaction network. The diamond shapes represented the herbs. The blue circles represented the number of the compound. The green and orange squares represented the chemical composition of Chenpi and Rougui, respectively. The red triangles represented the disease. The blue circles represented the overlapping targets between CRP-related targets and NAFLD-related targets, which are potential targets for CRP against NAFLD.


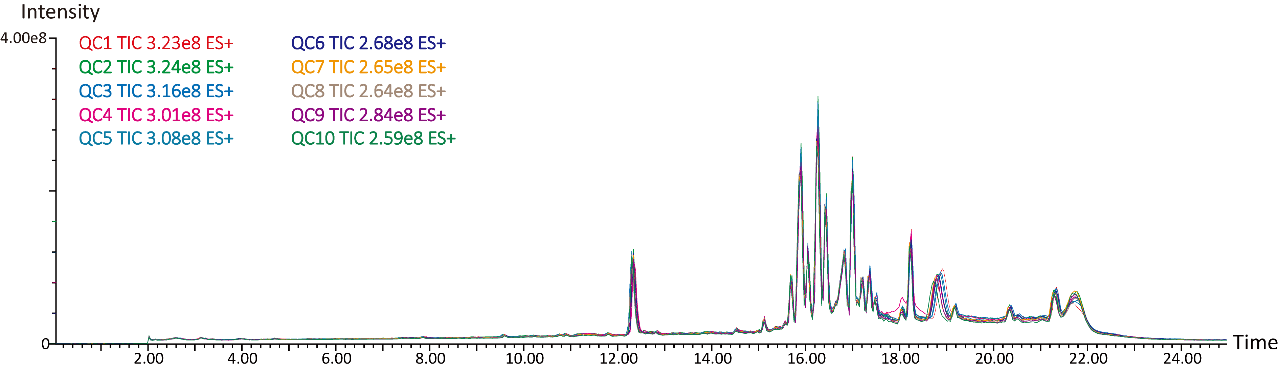


Figure S3. The total ion chromatogram (TIC) of QC samples using UPLC-QTOF-MS/MS. The y axis was the peak intensity, and the x axis was the retention time.


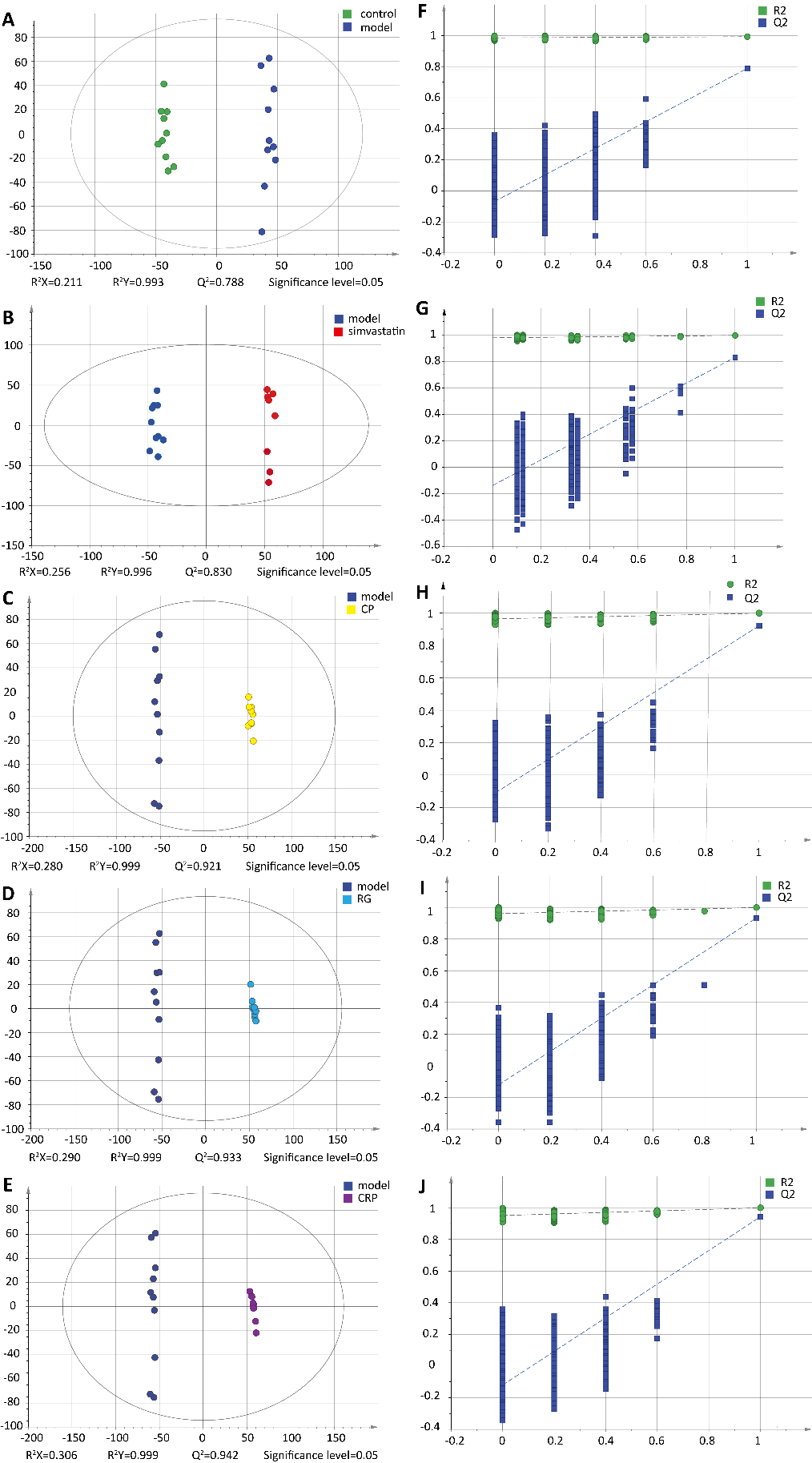


Figure S4. OPLS-DA (A-E) and 999-time permutation score plots (F-J) in metabolomics analysis. (A, F) control vs. model group, (B, G) model vs. simvastatin group, (C, H) model vs. CP group, (D, I): model vs. RG group, (E, J): model vs. CRP group.


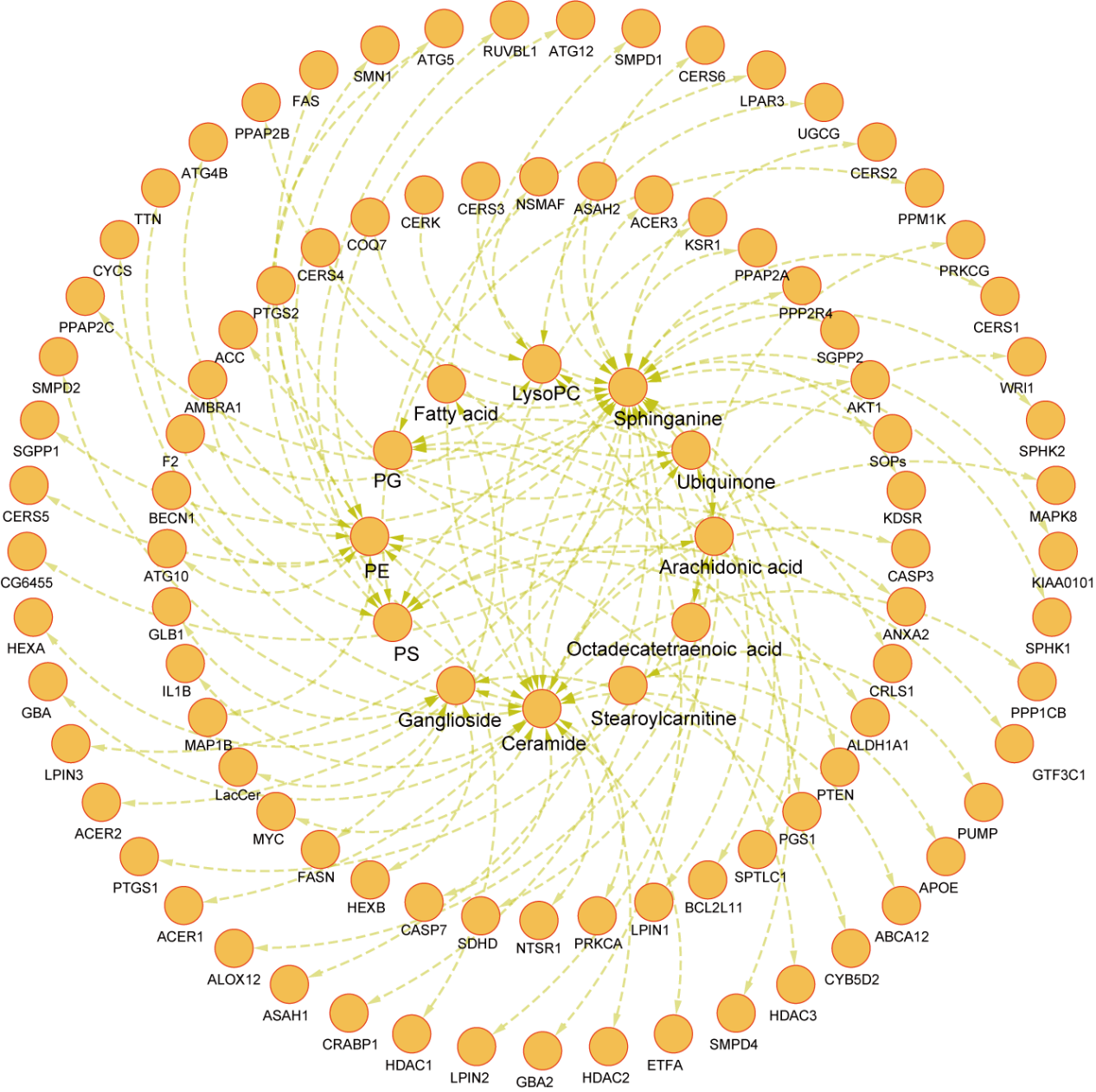


Figure S5. Differential metabolites related targets.

Table S1. The primer sequence of the targeted genes and internal reference gene.

| Gene | Sequence (5'-3') | Reverse (5'-3') | Target size (bp) |
| --- | --- | --- | --- |
| GAPDH | CCTCGTCCCGTAGACAAAATG | TGAGGTCAATGAAGGGGTCGT | 133 |
| PTGS2 | GAAATATCAGGTCATTGGTGGAGA | ATGCTCCTGCTTGAGTATGTCG | 205 |
| ALOX12 | GAGGATTCCCTGTCTCCTTCCA | GGAGGCATCCTCATTGTGC | 157 |
| HDAC1 | CACAAAGCCAATGCTGAGGAG | CGATGTCCGTCTGCTGCTTAT | 226 |
| CASP3 | TGGAATGTCATCTCGCTCTGGT | GAAGAGTTTCGGCTTTCCAGTC | 298 |
| AKT1 | CTTCCTCCTCAAGAACGATGGC | TGTCTTCATCAGCTGGCATTGT | 118 |
| F2 | GTCTGGAAGGTCGCTGTGCTAT | GAGTTGATTTCAGGCTTGTGGG | 124 |
| FASN | TGAATCAGCCCCACGCAGT | CCGAGTCAGTCTTGGAGGACAT | 297 |
| PRKCA | GGAAGTCTGTAGATTGGTGGGC | GGCAGGGTGTTTGGTCATAAGT | 188 |

Table S2. Identification of compounds in CRP based on UPLC-QTOF-MS/MS.

| No* | Name | Type^#^ | Formula | Mode | Precursor Ion (m/z) | Error (PPM) | Retention (min) | MS/MS (m/z) |
| --- | --- | --- | --- | --- | --- | --- | --- | --- |
| 1^b^ | glucose | P | C_6_H_12_O_6_ | negative | 179.0562 | 3.91 | 1.05 | 179.0549, 161.0452 |
| 2^a^ | pipecolic acid | A | C_6_H_11_NO_2_ | positive | 130.0856 | -9.22 | 1.09 | 84.0796, 82.0649 |
| 3^b^ | cinnamaldehyde | P | C_9_H_8_O | negative | 131.0485 | -8.39 | 1.09 | 103.0554 |
| 4^a^ | N-acetylnorsynephrine-rha | A | C_15_H_23_NO_6_ | positive | 314.1604 | 0.00 | 1.14 | 152.1049, 121.0559 |
| 5^b^ | citric acid | P | C_6_H_8_O_7_ | negative | 191.0213 | 11.52 | 1.21 | 111.0063 |
| 6^a^ | citrus D | A | C_12_H_15_NO_3_ | positive | 222.1145 | 6.75 | 1.52 | 143.0183, 119.0349 |
| 7^a^ | citrus L | A | C_25_H_22_N_2_O | positive | 367.1862 | 14.16 | 1.64 | 322.1385, 229.1008 |
| 8^a^ | N-acetylnorsynephrine | A | C_9_H_13_NO | positive | 152.1075 | 0.00 | 1.67 | 121.1631, 103.0540 |
| 9^a^ | adenosine | A | C_10_H_14_N_5_O_4_ | positive | 269.1092 | -11.89 | 1.71 | 136.0621, 119.0349 |
| 10^a^ | hydroxyadenosine | A | C_10_H_14_N_5_O_5_ | positive | 285.1066 | -2.46 | 1.92 | 152.0570, 135.0305 |
| 11^b^ | coumarin | T | C_6_H_10_O_4_ | negative | 145.0506 | 4.14 | 2.10 | 145.0485, 101.0587 |
| 12^b^ | syringic acid | P | C_9_H_10_O_5_ | negative | 197.0455 | 3.04 | 2.21 | 149.0239, 123.0450, 107.0488 |
| 13^a^ | citrus I | T | C_12_H_20_O_7_ | positive | 277.1274 | -4.69 | 2.23 | 211.0722, 133.0500 |
| 14^b^ | vinylacrylic acid-sophoroside | T | C_17_H_26_O_12_ | negative | 421.1328 | -4.27 | 3.16 | 195.0648, 145.0279 |
| 15^a^ | phenylalanine | A | C_9_H_12_NO_2_ | positive | 167.0962 | 9.57 | 3.22 | 131.0490, 120.0820, 103.0540 |
| 16^b^ | protocatechuic acid | P | C_7_H_6_O_4_ | negative | 153.0195 | 5.23 | 3.78 | 153.0179, 109.0277 |
| 17^b^ | protocatechuic acid hexoside | P | C_13_H_16_O_9_ | negative | 315.0691 | -7.93 | 3.81 | 315.0716, 153.0191, 109.0274 |
| 18^b^ | salidroside | P | C_13_H_16_O_8_ | negative | 299.077 | 1.34 | 4.74 | 178.0839, 137.0229 |
| 19^b^ | protocatechualdehyde | P | C_7_H_6_O_3_ | negative | 137.0237 | -0.73 | 6.14 | 137.0235, 119.0128 |
| 20^b^ | vanillin-glu | P | C_14_H_18_O_8_ | negative | 313.0912 | -3.51 | 6.45 | 153.0549, 123.0433 |
| 21^b^ | vanillic acid-glu | P | C_14_H_18_O_9_ | negative | 329.0885 | 3.95 | 6.98 | 167.0341, 153.0552, 109.0277 |
| 22^b^ | caffeoylglucose | P | C_15_H_18_O_9_ | negative | 341.0869 | -0.88 | 7.45 | 179.0349, 135.0447 |
| 23^b^ | hydroxy-junipediol A-glu | P | C_16_H_24_O_10_ | negative | 375.1281 | -2.67 | 7.57 | 301.0059, 197.0074 |
| 24^b^ | carboxylated gallocatechin | O | C_16_H_14_O_9_ | negative | 349.0599 | 11.46 | 8.00 | 349.0588, 305.0469, 165.0189 |
| 25^a^ | cyclocalamin | L | C_27_H_34_O_9_ | positive | 503.2269 | -2.38 | 8.78 | 125.0240 |
| 26^b^ | 3-O-caffeoylquinic acid | P | C_16_H_18_O_9_ | negative | 353.0886 | 3.97 | 9.62 | 191.0554, 179.0345, 135.0339 |
| 27^b^ | caffeic acid | P | C_9_H_8_O_4_ | negative | 179.0343 | -0.56 | 9.67 | 151.0388, 135.0442 |
| 28^b^ | cinncassiol B glu | T | C_26_H_42_O_13_ | negative | 561.2559 | 2.14 | 9.74 | 381.1914, 345.1688, 119.0344 |
| 29^a^ | trimethoxyflavone-ara | F | C_23_H_26_O_10_ | positive | 463.1599 | -1.08 | 10.05 | 185.1000, 153.0199 |
| 30^b^ | procyanidin B | O | C_30_H_26_O_12_ | negative | 577.1334 | -2.08 | 10.33 | 289.0725, 179.0342, 125.0235 |
| 31^b^ | glucosylhexanoic acid | P | C_12_H_22_O_8_ | negative | 293.122 | -5.46 | 10.98 | 131.0705, 113.0239 |
| 32^b^ | melittoside | T | C_21_H_32_O_15_ | negative | 523.1678 | 3.06 | 11.45 | 125.0239 |
| 33^b^ | gallocatechin | O | C_15_H_14_O_7_ | negative | 305.0668 | 2.29 | 11.48 | 261.0734, 137.0233, 109.0291 |
| 34^b^ | resorcinolic acid-ara-rha | P | C_18_H_24_O_12_ | negative | 431.1172 | -3.94 | 11.53 | 137.0235 |
| 35^b^ | catechin | O | C_15_H_14_O_6_ | negative | 289.0727 | 5.19 | 12.03 | 245.0816, 151.0394 |
| 36^a^ | tetrahydroxyflavone-glu-glu | F | C_27_H_30_O_17_ | positive | 627.1563 | 0.32 | 12.47 | 465.1039, 303.0506, 145.0492 |
| 37^b^ | cinnacasolide B | P | C_19_H_28_O_12_ | negative | 447.1527 | 5.59 | 12.57 | 161.0449, 149.0610 |
| 38^a^ | citrus G | A | C_10_H_13_NO_2_ | positive | 180.1036 | 6.11 | 13.00 | 163.1320, 107.0662 |
| 39^b^ | methoxy-spionoside A | T | C_20_H_32_O_10_ | negative | 431.1937 | 4.64 | 13.09 | 205.1233, 153.0911 |
| 40^b^ | dihydromelilotoside | P | C_15_H_20_O_8_ | negative | 327.1078 | -0.31 | 13.27 | 165.0549, 147.0451 |
| 41^b^ | alpinoside | T | C_18_H_24_O_11_ | negative | 415.1241 | 0.24 | 13.43 | 269.1022, 113.0237 |
| 42^a^ | luteolin-glu-rha | F | C_27_H_30_O_15_ | positive | 595.1676 | 2.18 | 14.27 | 287.0557, 147.0647 |
| 43^b^ | isoquercitrin 6-O-hydrocinnamic acid ester | F | C_30_H_28_O_13_ | negative | 595.1472 | 3.53 | 14.27 | 300.0276, 255.0306, 151.0028 |
| 44^a^ | naringin | F | C_27_H_32_O_14_ | positive | 581.188 | 1.72 | 14.79 | 435.1267, 273.0781, 155.0341, 119.0481 |
| 45^a^ | apigenin | F | C_15_H_10_O_5_ | positive | 271.0597 | -3.32 | 15.26 | 243.0618, 153.0199, 119.0483 |
| 46^b^ | dihydroxymethyl-benzoic acid | P | C_9_H_10_O_3_ | negative | 165.0541 | -6.06 | 15.26 | 165.0551, 121.0646 |
| 47^b^ | cinncassiol A glu | T | C_26_H_40_O_12_ | negative | 543.2443 | 0.37 | 15.51 | 363.1814, 167.1067 |
| 48^a^ | luteolin-glu | F | C_21_H_20_O_11_ | positive | 449.1098 | 3.12 | 15.52 | 287.0522, 147.0448, 129.0538 |
| 49^a^ | eriodictyol-glu | F | C_21_H_22_O_11_ | positive | 451.1198 | -9.31 | 15.57 | 153.0199, 107.0492 |
| 50^b^ | lyoniresinol-glu | C | C_28_H_38_O_13_ | negative | 581.2237 | 0.52 | 15.57 | 373.1288, 153.0545 |
| 51^b^ | quercetin 3-rha-ara | F | C_26_H_28_O_15_ | negative | 579.1373 | 4.14 | 15.64 | 300.0273, 255.0308, 151.0029 |
| 52^b^ | kaempferol 3-glu | F | C_21_H_20_O_11_ | negative | 447.0921 | -1.34 | 15.82 | 285.0384, 151.0029 |
| 53^b^ | cinnzeylanol | T | C_20_H_32_O_7_ | negative | 383.2065 | -1.04 | 16.03 | 383.2079, 243.1018 |
| 54^b^ | grandionoside A | T | C_19_H_32_O_8_ | negative | 387.2014 | -1.03 | 16.14 | 387.5216, 101.0233 |
| 55^a^ | hesperitin-glu-ara-rha | F | C_33_H_40_O_20_ | positive | 757.2151 | -5.28 | 16.22 | 449.1401, 129.0538 |
| 56^b^ | naringenin diglucoside | F | C_27_H_32_O_15_ | negative | 595.1691 | 4.87 | 16.38 | 385.0925, 313.0716, 271.0243 |
| 57^a^ | eriocitrin | F | C_27_H_32_O_15_ | positive | 597.1812 | -1.17 | 16.41 | 449.1358, 287.0522 |
| 58^a^ | eriodictyol | F | C_15_H_12_O_6_ | positive | 289.0701 | -3.81 | 16.45 | 163.0406, 153.0173, 145.0295 |
| 59^a^ | neohesperidin | F | C_28_H_34_O_15_ | positive | 611.1989 | 2.13 | 16.53 | 449.1445, 303.0862, 129.0561 |
| 60^a^ | 7α-obacunol | L | C_26_H_32_O_7_ | positive | 457.2206 | -4.37 | 16.60 | 329.0890, 177.0546 |
| 61^b^ | kaempferol-glu-ara | F | C_26_H_28_O_14_ | negative | 563.1397 | -0.53 | 17.00 | 284.0328, 151.0029 |
| 62^b^ | cinnamic acid | P | C_9_H_8_O_2_ | negative | 147.0452 | 4.08 | 17.03 | 147.0449 |
| 63^b^ | icariside B7 | T | C_25_H_42_O_11_ | negative | 517.2642 | -1.16 | 17.27 | 373.2241 |
| 64^b^ | melilotoside | P | C_15_H_18_O_8_ | negative | 325.0924 | 0.31 | 17.43 | 163.0389, 119.0488 |
| 65^b^ | aconiside | C | C_27_H_36_O_12_ | negative | 551.2056 | -10.06 | 17.50 | 389.1604, 151.1027 |
| 66^b^ | quercetin 3-glu | F | C_21_H_20_O_12_ | negative | 463.0843 | -7.13 | 17.53 | 301.0344, 155.1028 |
| 67^b^ | rutin | F | C_27_H_30_O_16_ | negative | 609.1483 | 4.6 | 17.59 | 300.0277, 178.9984, 151.0021 |
| 68^a^ | luteolin-glu-glu | F | C_27_H_30_O_16_ | positive | 611.1635 | 3.76 | 17.65 | 449.1098, 287.0522 |
| 69^a^ | narirutin | F | C_27_H_32_O_14_ | positive | 581.188 | 1.72 | 17.84 | 419.1331, 273.0781, 129.0538 |
| 70^a^ | trimethoxyflavone-rha | F | C_24_H_26_O_11_ | positive | 491.1606 | 10.79 | 18.00 | 345.0887 |
| 71^a^ | hesperitin | F | C_16_H_14_O_4_ | positive | 271.1001 | 11.44 | 18.12 | 177.0546, 145.0295 |
| 72^a^ | ichangensin | L | C_25_H_32_O_7_ | positive | 445.2197 | -6.51 | 18.31 | 303.0898, 153.0199 |
| 73^a^ | 7α-limonol | L | C_26_H_32_O_8_ | positive | 473.2193 | 3.8 | 18.55 | 219.0299, 115.0546 |
| 74^a^ | Hesperidin | F | C_28_H_34_O_15_ | positive | 611.1989 | 2.13 | 18.62 | 449.1445, 303.0862, 129.0538 |
| 75^a^ | apigenin-glu-O-rha | F | C_27_H_30_O_14_ | positive | 579.1721 | 1.21 | 18.67 | 271.0597, 153.0199, 129.0538 |
| 76^a^ | obacunone | L | C_26_H_30_O_7_ | positive | 455.2058 | -2.64 | 18.83 | 177.0546, 115.0546 |
| 77^a^ | bergapten | C | C_12_H_9_O_4_ | positive | 218.0598 | 8.71 | 18.98 | 202.0237, 174.0290, 131.0490 |
| 78^b^ | cassioside | T | C_20_H_32_O_9_ | negative | 415.195 | -4.34 | 19.14 | 309.9358, 213.9006 |
| 79^b^ | cinnzeylanine | T | C_22_H_34_O_8_ | negative | 425.2189 | 3.29 | 19.24 | 383.2084, 261.1136 |
| 80^b^ | methoxylhydroxylluteolin-glu | F | C_22_H_22_O_12_ | negative | 477.1051 | 3.77 | 19.36 | 315.0509, 299.0195, 151.0026 |
| 81^b^ | myo-inositol, 2-acetate 1, 6-dihexanoate | T | C_20_H_34_O_9_ | negative | 417.2128 | 0.96 | 19.45 | 239.4677, 219.2931 |
| 82^a^ | methyldeacetylnomilinate | L | C_27_H_36_O_9_ | positive | 505.2414 | -4.75 | 19.60 | 129.0537 |
| 83^b^ | cinncassins A4 | O | C_17_H_16_O_6_ | negative | 315.0872 | 1.27 | 19.63 | 271.0975, 256.0742 |
| 84^a^ | luteolin-glu-rha-O-glu | F | C_33_H_40_O_20_ | positive | 757.2151 | -5.28 | 19.83 | 611.1765, 287.0557 |
| 85^a^ | tryhydroxy-dymethoxyflavone-glu-rha | F | C_29_H_32_O_17_ | positive | 653.1712 | -0.92 | 19.86 | 347.0758, 129.0525 |
| 86^a^ | deoxylimonol | L | C_26_H_32_O_7_ | positive | 457.2238 | 2.62 | 20.29 | 129.0526 |
| 87^a^ | naringenin-ara-rha-ara | F | C_31_H_38_O_16_ | positive | 667.2255 | 2.55 | 21.00 | 521.1082, 273.0747 |
| 88^b^ | cinncassin A | O | C_16_H_14_O_5_ | negative | 285.0758 | -1.40 | 21.15 | 255.0672, 225.0577, 109.0274 |
| 89^a^ | poncirin | F | C_28_H_34_O_14_ | positive | 595.2026 | -0.17 | 21.22 | 449.1445, 287.0938, 129.0561 |
| 90^a^ | limonin | L | C_26_H_30_O_8_ | positive | 471.2006 | -2.76 | 21.41 | 425.1959, 375.1806, 347.1849 |
| 91^b^ | kaempferol 3-arabinoside | F | C_20_H_18_O_10_ | negative | 417.0832 | 2.64 | 21.77 | 285.0399, 227.0316 |
| 92^a^ | diosmetin-glu | F | C_22_H_22_O_11_ | positive | 463.1258 | 3.89 | 23.67 | 445.0229, 301.0732 |
| 93^a^ | nomilin | L | C_28_H_34_O_9_ | positive | 515.2252 | -5.63 | 23.70 | 453.1910, 193.0512, 125.0979 |
| 94^a^ | ichangin | L | C_26_H_32_O_9_ | positive | 489.2125 | 0.00 | 23.89 | 193.0513, 125.0980 |
| 95^b^ | dihydroxyoctane O-diglucoside | T | C_20_H_38_O_12_ | negative | 469.2298 | 2.77 | 24.76 | 291.1817, 101.0233 |
| 96^a^ | cirsimaritin-rha-ara-ara | F | C_33_H_40_O_18_ | positive | 725.228 | -1.79 | 24.95 | 315.0833, 129.0514 |
| 97^a^ | scopoletin | F | C_10_H_8_O_4_ | positive | 193.0483 | -9.32 | 25.38 | 179.0347, 151.0401, 159.0443, 131.0487, 103.0552 |
| 98^a^ | 3′, 4′, 6, 7-tetramethoxyflavone | F | C_19_H_18_O_6_ | positive | 343.1187 | 1.46 | 26.62 | 302.1536, 296.8699 |
| 99^a^ | 4′, 5, 6, 7-tetramethoxyflavone | F | C_19_H_18_O_6_ | positive | 343.1187 | 1.46 | 27.24 | 328.0875, 313.0706, 285.0760 |
| 100^a^ | trimethoxyflavone | F | C_18_H_16_O_7_ | positive | 345.0963 | -3.19 | 27.36 | 303.0826, 153.0173 |
| 101^b^ | 4-(3, 5-dihydroxy-7-(4-hydroxy-3-methoxyphenyl)heptyl)benzene-1, 2-diol | C | C_20_H_26_O_6_ | negative | 361.1609 | -11.63 | 28.39 | 277.0866, 233.0967, 115.1027 |
| 102^a^ | methoxyflavanone-ara | F | C_21_H_22_O_9_ | positive | 419.1331 | -2.62 | 28.45 | 383.1142, 285.0726 |
| 103^a^ | hydroxyl-pentamethoxyflavone | F | C_20_H_20_O_8_ | positive | 389.1214 | -5.65 | 28.82 | 374.0960, 359.0625, 256.0865 |
| 104^a^ | tangeretin | F | C_20_H_20_O_7_ | positive | 373.131 | 6.16 | 29.17 | 358.1031, 343.0808, 325.0700 |
| 105^a^ | auraptene | C | C_19_H_22_O_3_ | positive | 299.1625 | -7.35 | 31.70 | 163.0379, 137.1335 |

*: ^a^ the compoundsPdentified from Chenpi; ^b^ the compoundsPdentified from Rougui.

#: P, phenolic acids (aldehyde); A, alkaloids; T, terpenoids; L, limonoids; F, flavonoids ; C, coumarins ; O, others.

Table S3. Compounds of CRP related targets.

| Uniprot ID | Component | Targets | Probability* |
| --- | --- | --- | --- |
| P43166 | eriodictyol | CA7 | 1.0000 |
| O43570 | eriodictyol | CA12 | 1.0000 |
| P22748 | eriodictyol | CA4 | 1.0000 |
| Q16678 | eriodictyol | CYP1B1 | 1.0000 |
| P11511 | naringin | CYP19A1 | 1.0000 |
| Q9NPH5 | apigenin | NOX4 | 1.0000 |
| P15121 | apigenin | AKR1B1 | 1.0000 |
| Q15078 | apigenin | CDK5R1 | 1.0000 |
| Q15078 | apigenin | CDK5 | 1.0000 |
| P47989 | apigenin | XDH | 1.0000 |
| P21397 | apigenin | MAOA | 1.0000 |
| P36888 | apigenin | FLT3 | 1.0000 |
| P11511 | apigenin | CYP19A1 | 1.0000 |
| P03372 | apigenin | ESR1 | 1.0000 |
| Q8WWL7 | apigenin | CCNB3 | 1.0000 |
| P22303 | apigenin | ACHE | 1.0000 |
| P30542 | apigenin | ADORA1 | 1.0000 |
| P35354 | apigenin | PTGS2 | 1.0000 |
| Q92731 | apigenin | ESR2 | 1.0000 |
| Q00534 | apigenin | CDK6 | 1.0000 |
| P29274 | apigenin | ADORA2A | 1.0000 |
| P43405 | apigenin | SYK | 1.0000 |
| P49841 | apigenin | GSK3B | 1.0000 |
| P33527 | apigenin | ABCC1 | 1.0000 |
| P14061 | apigenin | HSD17B1 | 1.0000 |
| P02766 | apigenin | TTR | 1.0000 |
| P68400 | apigenin | CSNK2A1 | 1.0000 |
| P13569 | apigenin | CFTR | 1.0000 |
| Q16678 | apigenin | CYP1B1 | 1.0000 |
| Q9UNQ0 | apigenin | ABCG2 | 1.0000 |
| O60218 | apigenin | AKR1B10 | 1.0000 |
| Q9H2K2 | apigenin | TNKS2 | 1.0000 |
| O95271 | apigenin | TNKS | 1.0000 |
| P43166 | hesperitin | CA7 | 1.0000 |
| O43570 | hesperitin | CA12 | 1.0000 |
| P22748 | hesperitin | CA4 | 1.0000 |
| Q16678 | hesperitin | CYP1B1 | 1.0000 |
| P41143 | tangeretin | OPRD1 | 1.0000 |
| P21397 | auraptene | MAOA | 1.0000 |
| P27338 | auraptene | MAOB | 1.0000 |
| P00918 | syringic acid | CA2 | 1.0000 |
| P43166 | syringic acid | CA7 | 1.0000 |
| P00915 | syringic acid | CA1 | 1.0000 |
| P07451 | syringic acid | CA3 | 1.0000 |
| P23280 | syringic acid | CA6 | 1.0000 |
| O43570 | syringic acid | CA12 | 1.0000 |
| Q9ULX7 | syringic acid | CA14 | 1.0000 |
| Q16790 | syringic acid | CA9 | 1.0000 |
| P35218 | syringic acid | CA5A | 1.0000 |
| P21836 | rutin | Ache | 1.0000 |
| P06493 | apigenin | CDK1 | 1.0000 |
| P14635 | apigenin | CCNB1 | 1.0000 |
| O75762 | cinnamaldehyde | TRPA1 | 0.9995 |
| P30542 | adenosine | ADORA1 | 0.9953 |
| P29274 | adenosine | ADORA2A | 0.9953 |
| P0DMS8 | adenosine | ADORA3 | 0.9953 |
| P43166 | scopoletin | CA7 | 0.9888 |
| O43570 | scopoletin | CA12 | 0.9888 |
| Q16790 | scopoletin | CA9 | 0.9888 |
| P27487 | adenosine | DPP4 | 0.9639 |
| P55263 | adenosine | ADK | 0.9481 |
| P00918 | protocatechuic acid | CA2 | 0.9263 |
| P43166 | protocatechuic acid | CA7 | 0.9263 |
| P00915 | protocatechuic acid | CA1 | 0.9263 |
| P23280 | protocatechuic acid | CA6 | 0.9263 |
| O43570 | protocatechuic acid | CA12 | 0.9263 |
| Q9ULX7 | protocatechuic acid | CA14 | 0.9263 |
| Q16790 | protocatechuic acid | CA9 | 0.9263 |
| P22748 | protocatechuic acid | CA4 | 0.9263 |
| P11511 | narirutin | CYP19A1 | 0.8930 |
| Q9EP66 | cinnamic acid | Hcar2 | 0.8870 |
| P00918 | coumarin | CA2 | 0.8572 |
| P43166 | coumarin | CA7 | 0.8572 |
| P00915 | coumarin | CA1 | 0.8572 |
| P07451 | coumarin | CA3 | 0.8572 |
| P23280 | coumarin | CA6 | 0.8572 |
| O43570 | coumarin | CA12 | 0.8572 |
| Q9ULX7 | coumarin | CA14 | 0.8572 |
| Q16790 | coumarin | CA9 | 0.8572 |
| P22748 | coumarin | CA4 | 0.8572 |
| Q8N1Q1 | coumarin | CA13 | 0.8572 |
| Q9Y2D0 | coumarin | CA5B | 0.8572 |
| P35218 | coumarin | CA5A | 0.8572 |
| P00918 | caffeic acid | CA2 | 0.7393 |
| P09917 | caffeic acid | ALOX5 | 0.7393 |
| P43166 | caffeic acid | CA7 | 0.7393 |
| P00915 | caffeic acid | CA1 | 0.7393 |
| P23280 | caffeic acid | CA6 | 0.7393 |
| P14780 | caffeic acid | MMP9 | 0.7393 |
| O43570 | caffeic acid | CA12 | 0.7393 |
| P03956 | caffeic acid | MMP1 | 0.7393 |
| P08253 | caffeic acid | MMP2 | 0.7393 |
| P18031 | caffeic acid | PTPN1 | 0.7393 |
| Q9ULX7 | caffeic acid | CA14 | 0.7393 |
| Q16790 | caffeic acid | CA9 | 0.7393 |
| Q9Y2D0 | caffeic acid | CA5B | 0.7393 |
| P35218 | caffeic acid | CA5A | 0.7393 |
| P15121 | 3-O-caffeoylquinic acid | AKR1B1 | 0.6998 |
| P11511 | eriodictyol | CYP19A1 | 0.6498 |
| P09917 | apigenin | ALOX5 | 0.6003 |
| P09874 | apigenin | PARP1 | 0.6003 |
| O60218 | 3-O-caffeoylquinic acid | AKR1B10 | 0.5910 |
| P08253 | procyanidin B or its isomers | MMP2 | 0.5773 |
| P00918 | apigenin | CA2 | 0.5590 |
| P43166 | apigenin | CA7 | 0.5590 |
| O43570 | apigenin | CA12 | 0.5590 |
| P08183 | apigenin | ABCB1 | 0.5590 |
| Q8N1Q1 | scopoletin | CA13 | 0.4917 |
| P22748 | apigenin | CA4 | 0.4855 |
| Q9ERQ8 | rutin | Ca7 | 0.4780 |
| P18054 | apigenin | ALOX12 | 0.4774 |
| P16050 | auraptene | ALOX15 | 0.4591 |
| P01375 | luteolin-O-glucoside-O-glucoside | TNF | 0.4302 |
| P60568 | luteolin-O-glucoside-O-glucoside | IL2 | 0.4302 |
| P01375 | luteolin-O-glucoside | TNF | 0.4302 |
| P60568 | luteolin-O-glucoside | IL2 | 0.4302 |
| Q13332 | apigenin | PTPRS | 0.4119 |
| P47989 | luteolin-O-glucoside-O-glucoside | XDH | 0.4109 |
| P15121 | luteolin-O-glucoside | AKR1B1 | 0.3970 |
| P01375 | acacetin-O-glucoside | TNF | 0.3970 |
| P11511 | neohesperidin | CYP19A1 | 0.3921 |
| P47989 | luteolin-O-glucoside-orhamnoside/apigenin-6,8-di-C-glucosid | XDH | 0.3883 |
| P11511 | eriocitrin | CYP19A1 | 0.3883 |
| P11511 | poncirin | CYP19A1 | 0.3795 |
| P01375 | diosmetin-O-glucoside | TNF | 0.3772 |
| P60568 | diosmetin-O-glucoside | IL2 | 0.3772 |
| Q04760 | apigenin | GLO1 | 0.3709 |
| P05067 | apigenin | APP | 0.3709 |
| P14780 | apigenin | MMP9 | 0.3709 |
| P08253 | apigenin | MMP2 | 0.3709 |
| P39900 | apigenin | MMP12 | 0.3709 |
| P28907 | apigenin | CD38 | 0.3709 |
| P11387 | apigenin | TOP1 | 0.3709 |
| P05089 | apigenin | ARG1 | 0.3709 |
| P15121 | tangeretin | AKR1B1 | 0.3613 |
| P11474 | apigenin | ESRRA | 0.3545 |
| P49763 | procyanidin B or its isomers | PGF | 0.3525 |
| P15692 | procyanidin B or its isomers | VEGFA | 0.3525 |
| P01375 | 3′,4′,5′,5-hydroxy-flavone-O-glucoside-O-glucoside | TNF | 0.3511 |
| P60568 | 3′,4′,5′,5-hydroxy-flavone-O-glucoside-O-glucoside | IL2 | 0.3511 |
| P47989 | 3′,4′,5′,5-hydroxy-flavone-O-glucoside-O-glucoside | XDH | 0.3321 |
| P11142 | adenosine | HSPA8 | 0.3320 |
| P11021 | adenosine | HSPA5 | 0.3320 |
| Q16875 | apigenin | PFKFB3 | 0.3299 |
| P16050 | apigenin | ALOX15 | 0.3299 |
| P04745 | apigenin | AMY1A | 0.3299 |
| P43250 | apigenin | GRK6 | 0.3299 |
| P60568 | 3′,4′,5′-trimethoxyflavone-O-rhamnoside | IL2 | 0.3288 |
| P00813 | adenosine | ADA | 0.3242 |
| P30542 | hydroxyadenosine | ADORA1 | 0.3217 |
| P29274 | hydroxyadenosine | ADORA2A | 0.3217 |
| P0DMS8 | hydroxyadenosine | ADORA3 | 0.3217 |
| P01375 | luteolin-O-glucoside-Orhamnoside/apigenin-6,9-di-C-glucosid | TNF | 0.2983 |
| P60568 | luteolin-O-glucoside-orhamnoside/apigenin-6,10-di-C-glucosid | IL2 | 0.2983 |
| P11511 | hesperidin | CYP19A1 | 0.2960 |
| P23219 | eriodictyol | PTGS1 | 0.2955 |
| P53396 | citric acid | ACLY | 0.2915 |
| P27487 | hydroxyadenosine | DPP4 | 0.2889 |
| P47989 | 3′,3,5-hydroxy-4′,5′-methoxyflavone-O-glucoside-orhamnoside | XDH | 0.2803 |
| P14679 | apigenin | TYR | 0.2726 |
| P37059 | apigenin | HSD17B2 | 0.2726 |
| P35869 | apigenin | AHR | 0.2726 |
| P60568 | acacetin-O-glucoside | IL2 | 0.2725 |
| P23526 | adenosine | AHCY | 0.2691 |
| P04406 | adenosine | GAPDH | 0.2691 |
| P35372 | tangeretin | OPRM1 | 0.2572 |
| P55263 | hydroxyadenosine | ADK | 0.2562 |
| P00915 | apigenin | CA1 | 0.2562 |
| Q16790 | apigenin | CA9 | 0.2562 |
| P08908 | dihydroxy-7-(4-hydroxy-3-methoxyphenyl)heptyl]-benzenediol | HTR1A | 0.2554 |
| P11511 | naringenin-O-arabinoside-O-rhamnoside-O-arabinoside | CYP19A1 | 0.2495 |
| P16152 | apigenin | CBR1 | 0.2479 |
| P10275 | apigenin | AR | 0.2479 |
| O14746 | apigenin | TERT | 0.2396 |
| P49810 | cinncassiol b glucoside | PSEN2 | 0.2373 |
| P11309 | apigenin | PIM1 | 0.2316 |
| P18654 | rutin | Rps6ka3 | 0.2290 |
| P07900 | dihydroxyoctane O-diglucoside | HSP90AA1 | 0.2227 |
| P30542 | acacetin-O-glucoside | ADORA1 | 0.2227 |
| P41145 | auraptene | OPRK1 | 0.2220 |
| P41145 | limonin | OPRK1 | 0.2200 |
| P41143 | limonin | OPRD1 | 0.2200 |
| Q9UNQ0 | tangeretin | ABCG2 | 0.2173 |
| P15692 | dihydroxyoctane O-diglucoside | VEGFA | 0.2144 |
| P05230 | dihydroxyoctane O-diglucoside | FGF1 | 0.2144 |
| Q9Y251 | dihydroxyoctane O-diglucoside | HPSE | 0.2144 |
| P01375 | luteolin-O-glucoside-O-rhamnoside-O-glucoside | TNF | 0.2135 |
| P60568 | luteolin-O-glucoside-O-rhamnoside-O-glucoside | IL2 | 0.2135 |
| P07900 | cinncassiol B glucoside | HSP90AA1 | 0.2126 |
| P07451 | caffeic acid | CA3 | 0.2124 |
| P14780 | procyanidin B or its isomers | MMP9 | 0.2082 |
| P14416 | N-acetylnorsynephrine | DRD2 | 0.2070 |
| P11511 | hesperitin | CYP19A1 | 0.2063 |
| P33527 | eriodictyol | ABCC1 | 0.2049 |
| P14061 | eriodictyol | HSD17B1 | 0.2049 |
| P04278 | eriodictyol | SHBG | 0.2049 |
| P16152 | eriodictyol | CBR1 | 0.2049 |
| Q92731 | eriodictyol | ESR2 | 0.2049 |
| P00533 | apigenin | EGFR | 0.1988 |
| P15121 | 3′,4′,5′-trimethoxyflavone-O-rhamnoside | AKR1B1 | 0.1979 |
| P03372 | cinncassin A | ESR1 | 0.1967 |
| Q92731 | cinncassin A | ESR2 | 0.1967 |
| P41145 | cyclocalamin | OPRK1 | 0.1960 |
| P41145 | nomilin | OPRK1 | 0.1917 |
| P06493 | apigenin | CDK1 | 0.1907 |
| P03372 | eriodictyol | ESR1 | 0.1884 |
| P01375 | apigenin-O-glucoside-O-rhamnoside | TNF | 0.1812 |
| P30542 | 3′,4′,5′-trimethoxyflavone-O-rhamnoside | ADORA1 | 0.1805 |
| P15121 | diosmetin-O-glucoside | AKR1B1 | 0.1761 |
| P41143 | nomilin | OPRD1 | 0.1752 |
| Q9H9B1 | adenosine | EHMT1 | 0.1749 |
| Q96KQ7 | adenosine | EHMT2 | 0.1749 |
| P47989 | luteolin-O-glucoside-O-rhamnoside-O-glucoside | XDH | 0.1738 |
| P11511 | eriodictyol-O-glucoside | CYP19A1 | 0.1729 |
| P11511 | naringenin diglucoside | CYP19A1 | 0.1723 |
| P41145 | ichangin | OPRK1 | 0.1717 |
| Q16678 | tangeretin | CYP1B1 | 0.1693 |
| Q96RJ0 | N-acetylnorsynephrine | TAAR1 | 0.1684 |
| P59538 | hesperitin | TAS2R31 | 0.1668 |
| P30542 | hesperitin | ADORA1 | 0.1668 |
| P0DMS8 | hesperitin | ADORA3 | 0.1668 |
| Q01650 | phenylalanine | SLC7A5 | 0.1666 |
| B2RXH2 | apigenin | KDM4E | 0.1661 |
| P15121 | luteolin-O-glucoside-orhamnoside/apigenin-6,11-di-C-glucosid | AKR1B1 | 0.1633 |
| P41145 | methyl deacetylnomilinate | OPRK1 | 0.1623 |
| P10721 | tangeretin | KIT | 0.1613 |
| P30542 | diosmetin-O-glucoside | ADORA1 | 0.1593 |
| Q9UNQ0 | hesperitin | ABCG2 | 0.1589 |
| P14061 | hesperitin | HSD17B1 | 0.1589 |
| P35462 | N-acetylnorsynephrine | DRD3 | 0.1585 |
| O75899 | phenylalanine | GABBR2 | 0.1584 |
| P30542 | luteolin-O-glucoside | ADORA1 | 0.1563 |
| P35372 | limonin | OPRM1 | 0.1535 |
| P35354 | tangeretin | PTGS2 | 0.1533 |
| Q92731 | hesperitin | ESR2 | 0.1510 |
| P56817 | auraptene | BACE1 | 0.1510 |
| P15121 | caffeic acid | AKR1B1 | 0.1509 |
| Q92731 | caffeic acid | ESR2 | 0.1509 |
| P22748 | caffeic acid | CA4 | 0.1509 |
| P06239 | apigenin | LCK | 0.1497 |
| P49810 | dihydroxyoctane O-diglucoside | PSEN2 | 0.1480 |
| P27338 | Eriodictyol | MAOB | 0.1473 |
| P01375 | 3′,4′,5′-trimethoxyflavone-O-rhamnoside | TNF | 0.1455 |
| P00915 | Scopoletin | CA1 | 0.1438 |
| O43570 | Auraptene | CA12 | 0.1431 |
| Q16790 | Auraptene | CA9 | 0.1431 |
| P15121 | luteolin-O-glucoside-O-glucoside | AKR1B1 | 0.1428 |
| P35372 | nomilin | OPRM1 | 0.1422 |
| P0DMS8 | phenylalanine | ADORA3 | 0.1418 |
| B2RXH2 | phenylalanine | KDM4E | 0.1418 |
| P06241 | phenylalanine | FYN | 0.1418 |
| P00533 | phenylalanine | EGFR | 0.1418 |
| Q96GD4 | apigenin | AURKB | 0.1415 |
| Q13564 | apigenin | NAE1 | 0.1415 |
| P24557 | apigenin | TBXAS1 | 0.1415 |
| P09038 | dihydroxyoctane O-diglucoside | FGF2 | 0.1397 |
| P45452 | 3-O-caffeoylquinic acid | MMP13 | 0.1391 |
| P08253 | 3-O-caffeoylquinic acid | MMP2 | 0.1391 |
| P59538 | eriodictyol | TAS2R31 | 0.1391 |
| P30542 | eriodictyol | ADORA1 | 0.1391 |
| P0DMS8 | eriodictyol | ADORA3 | 0.1391 |
| P35228 | tangeretin | NOS2 | 0.1373 |
| P29274 | tangeretin | ADORA2A | 0.1373 |
| P30542 | tangeretin | ADORA1 | 0.1373 |
| P0DMS8 | tangeretin | ADORA3 | 0.1373 |
| P41143 | ichangin | OPRD1 | 0.1368 |
| Q07820 | adenosine | MCL1 | 0.1356 |
| Q8WTS6 | adenosine | SETD7 | 0.1356 |
| P03372 | hesperitin | ESR1 | 0.1352 |
| P27338 | hesperitin | MAOB | 0.1352 |
| Q9UBS5 | phenylalanine | GABBR1 | 0.1334 |
| P04062 | glucose | GBA | 0.1334 |
| P07900 | glucose | HSP90AA1 | 0.1334 |
| P15692 | glucose | VEGFA | 0.1334 |
| P49810 | glucose | PSEN2 | 0.1334 |
| P05230 | glucose | FGF1 | 0.1334 |
| P09038 | glucose | FGF2 | 0.1334 |
| Q9Y251 | glucose | HPSE | 0.1334 |
| P11142 | hydroxyadenosine | HSPA8 | 0.1333 |
| P11021 | hydroxyadenosine | HSPA5 | 0.1333 |
| P08069 | apigenin | IGF1R | 0.1333 |
| P41145 | ichangensin | OPRK1 | 0.1314 |
| P15121 | acacetin-O-glucoside | AKR1B1 | 0.1314 |
| P17252 | myo-inositol, 2-acetate 1,6-dihexanoate | PRKCA | 0.1308 |
| P05067 | 3-O-caffeoylquinic acid | APP | 0.1307 |
| P15692 | cinncassiol B glucoside | VEGFA | 0.1302 |
| P05230 | cinncassiol B glucoside | FGF1 | 0.1302 |
| Q9Y251 | cinncassiol B glucoside | HPSE | 0.1302 |
| P47989 | apigenin-O-glucoside-O-rhamnoside | XDH | 0.1279 |
| P60568 | apigenin-O-glucoside-O-rhamnoside | IL2 | 0.1279 |
| P17707 | adenosine | AMD1 | 0.1278 |
| P00533 | adenosine | EGFR | 0.1278 |
| P33527 | hesperitin | ABCC1 | 0.1273 |
| P04278 | hesperitin | SHBG | 0.1273 |
| P16152 | hesperitin | CBR1 | 0.1273 |
| P45452 | hesperitin | MMP13 | 0.1273 |
| P28482 | nomilin | MAPK1 | 0.1257 |
| P56373 | nomilin | P2RX3 | 0.1257 |
| P21554 | nomilin | CNR1 | 0.1257 |
| P34972 | nomilin | CNR2 | 0.1257 |
| P21730 | nomilin | C5AR1 | 0.1257 |
| Q08881 | nomilin | ITK | 0.1257 |
| P18031 | nomilin | PTPN1 | 0.1257 |
| P54760 | nomilin | EPHB4 | 0.1257 |
| P32246 | nomilin | CCR1 | 0.1257 |
| P53671 | nomilin | LIMK2 | 0.1257 |
| P07333 | nomilin | CSF1R | 0.1257 |
| Q5S007 | nomilin | LRRK2 | 0.1257 |
| P17706 | nomilin | PTPN2 | 0.1257 |
| P42345 | nomilin | MTOR | 0.1257 |
| Q13547 | nomilin | HDAC1 | 0.1257 |
| P56524 | nomilin | HDAC4 | 0.1257 |
| P42336 | nomilin | PIK3CA | 0.1257 |
| P20309 | nomilin | CHRM3 | 0.1257 |
| Q8NER1 | nomilin | TRPV1 | 0.1257 |
| P56817 | nomilin | BACE1 | 0.1257 |
| P24864 | nomilin | CCNE1 | 0.1257 |
| P24941 | nomilin | CDK2 | 0.1257 |
| P49810 | nomilin | PSEN2 | 0.1257 |
| P34913 | nomilin | EPHX2 | 0.1257 |
| Q07817 | nomilin | BCL2L1 | 0.1257 |
| P49841 | nomilin | GSK3B | 0.1257 |
| P49840 | nomilin | GSK3A | 0.1257 |
| Q15078 | nomilin | CDK5R1 | 0.1257 |
| Q13627 | nomilin | DYRK1A | 0.1257 |
| P61586 | nomilin | RHOA | 0.1257 |
| P56704 | nomilin | WNT3A | 0.1257 |
| O75874 | nomilin | IDH1 | 0.1257 |
| Q9H2K2 | nomilin | TNKS2 | 0.1257 |
| P09874 | nomilin | PARP1 | 0.1257 |
| O76083 | nomilin | PDE9A | 0.1257 |
| Q99572 | nomilin | P2RX7 | 0.1257 |
| O15055 | nomilin | PER2 | 0.1257 |
| P28845 | nomilin | HSD11B1 | 0.1257 |
| P42785 | nomilin | PRCP | 0.1257 |
| P04035 | nomilin | HMGCR | 0.1257 |
| O14746 | nomilin | TERT | 0.1257 |
| Q9Y233 | nomilin | PDE10A | 0.1257 |
| P43490 | nomilin | NAMPT | 0.1257 |
| P25101 | nomilin | EDNRA | 0.1257 |
| Q16539 | nomilin | MAPK14 | 0.1257 |
| P15056 | nomilin | BRAF | 0.1257 |
| P17655 | nomilin | CAPN2 | 0.1257 |
| P43405 | nomilin | SYK | 0.1257 |
| P43235 | nomilin | CTSK | 0.1257 |
| P25774 | nomilin | CTSS | 0.1257 |
| Q16853 | nomilin | AOC3 | 0.1257 |
| P07711 | nomilin | CTSL | 0.1257 |
| P07384 | nomilin | CAPN1 | 0.1257 |
| P25116 | nomilin | F2R | 0.1257 |
| P00533 | nomilin | EGFR | 0.1257 |
| P20701 | nomilin | ITGAL | 0.1257 |
| P50750 | nomilin | CDK9 | 0.1257 |
| P49327 | nomilin | FASN | 0.1257 |
| P42330 | nomilin | AKR1C3 | 0.1257 |
| P00338 | nomilin | LDHA | 0.1257 |
| P04150 | nomilin | NR3C1 | 0.1257 |
| P00746 | nomilin | CFD | 0.1257 |
| P06737 | nomilin | PYGL | 0.1257 |
| P03956 | nomilin | MMP1 | 0.1257 |
| Q16850 | nomilin | CYP51A1 | 0.1257 |
| P30559 | nomilin | OXTR | 0.1257 |
| O60911 | nomilin | CTSV | 0.1257 |
| O60885 | nomilin | BRD4 | 0.1257 |
| Q15059 | nomilin | BRD3 | 0.1257 |
| P37288 | nomilin | AVPR1A | 0.1257 |
| P12268 | nomilin | IMPDH2 | 0.1257 |
| P25025 | nomilin | CXCR2 | 0.1257 |
| Q05469 | nomilin | LIPE | 0.1257 |
| Q99500 | nomilin | S1PR3 | 0.1257 |
| P25024 | nomilin | CXCR1 | 0.1257 |
| P53609 | nomilin | PGGT1B | 0.1257 |
| O60760 | nomilin | HPGDS | 0.1257 |
| P02766 | dihydroxy-7-(4-hydroxy-3-methoxyphenyl)heptyl]-benzenediol | TTR | 0.1256 |
| P35968 | apigenin | KDR | 0.1251 |
| P53350 | apigenin | PLK1 | 0.1251 |
| P08581 | apigenin | MET | 0.1251 |
| Q9UM73 | apigenin | ALK | 0.1251 |
| P30530 | apigenin | AXL | 0.1251 |
| P06276 | apigenin | BCHE | 0.1251 |
| P0DMS8 | apigenin | ADORA3 | 0.1251 |
| P24941 | apigenin | CDK2 | 0.1251 |
| P28335 | apigenin | HTR2C | 0.1251 |
| P25103 | phenylalanine | TACR1 | 0.1251 |
| P07101 | phenylalanine | TH | 0.1251 |
| P06239 | phenylalanine | LCK | 0.1251 |
| Q9H3R0 | phenylalanine | KDM4C | 0.1251 |
| P08913 | phenylalanine | ADRA2A | 0.1251 |
| P18089 | phenylalanine | ADRA2B | 0.1251 |
| P35348 | phenylalanine | ADRA1A | 0.1251 |
| P06493 | glucose | CDK1 | 0.1251 |
| P56470 | glucose | LGALS4 | 0.1251 |
| P17931 | glucose | LGALS3 | 0.1251 |
| O00214 | glucose | LGALS8 | 0.1251 |
| P30542 | luteolin-O-glucoside-O-glucoside | ADORA1 | 0.1236 |
| P15121 | 3′,4′,5′,5-hydroxy-flavone-O-glucoside-O-glucoside | AKR1B1 | 0.1224 |
| P00734 | cinncassiol A glucoside | F2 | 0.1219 |
| P18031 | cinncassiol A glucoside | PTPN1 | 0.1219 |
| O75688 | cinncassiol A glucoside | PPM1B | 0.1219 |
| Q15172 | cinncassiol A glucoside | PPP2R5A | 0.1219 |
| P05412 | cinncassiol A glucoside | JUN | 0.1219 |
| P17252 | cinncassiol A glucoside | PRKCA | 0.1219 |
| P08151 | cinncassiol A glucoside | GLI1 | 0.1219 |
| P0DMS8 | cinncassiol A glucoside | ADORA3 | 0.1219 |
| Q02156 | cinncassiol A glucoside | PRKCE | 0.1219 |
| P24723 | cinncassiol A glucoside | PRKCH | 0.1219 |
| Q04759 | cinncassiol A glucoside | PRKCQ | 0.1219 |
| P40763 | cinncassiol A glucoside | STAT3 | 0.1219 |
| Q07817 | cinncassiol A glucoside | BCL2L1 | 0.1219 |
| P29274 | cinncassiol A glucoside | ADORA2A | 0.1219 |
| P31639 | cinncassiol A glucoside | SLC5A2 | 0.1219 |
| P45452 | cinncassiol A glucoside | MMP13 | 0.1219 |
| P03956 | cinncassiol A glucoside | MMP1 | 0.1219 |
| P09237 | cinncassiol A glucoside | MMP7 | 0.1219 |
| P39900 | cinncassiol A glucoside | MMP12 | 0.1219 |
| P22894 | cinncassiol A glucoside | MMP8 | 0.1219 |
| P13866 | cinncassiol A glucoside | SLC5A1 | 0.1219 |
| Q99808 | cinncassiol A glucoside | SLC29A1 | 0.1219 |
| P01112 | cinncassiol A glucoside | HRAS | 0.1219 |
| P36873 | cinncassiol A glucoside | PPP1CC | 0.1219 |
| P11387 | cinncassiol A glucoside | TOP1 | 0.1219 |
| P14679 | cinncassiol A glucoside | TYR | 0.1219 |
| P05023 | cinncassiol A glucoside | ATP1A1 | 0.1219 |
| P05129 | cinncassiol A glucoside | PRKCG | 0.1219 |
| P08473 | cinncassiol A glucoside | MME | 0.1219 |
| P15498 | cinncassiol A glucoside | VAV1 | 0.1219 |
| Q9HBA0 | cinncassiol A glucoside | TRPV4 | 0.1219 |
| P06241 | cinncassiol A glucoside | FYN | 0.1219 |
| P42336 | cinncassiol A glucoside | PIK3CA | 0.1219 |
| P11388 | cinncassiol A glucoside | TOP2A | 0.1219 |
| P04626 | cinncassiol A glucoside | ERBB2 | 0.1219 |
| P00533 | cinncassiol A glucoside | EGFR | 0.1219 |
| P08253 | cinncassiol A glucoside | MMP2 | 0.1219 |
| O14965 | cinncassiol A glucoside | AURKA | 0.1219 |
| P05771 | cinncassiol A glucoside | PRKCB | 0.1219 |
| P00519 | cinncassiol A glucoside | ABL1 | 0.1219 |
| P29317 | cinncassiol A glucoside | EPHA2 | 0.1219 |
| P06239 | cinncassiol A glucoside | LCK | 0.1219 |
| P12931 | cinncassiol A glucoside | SRC | 0.1219 |
| P80192 | cinncassiol A glucoside | MAP3K9 | 0.1219 |
| P55263 | cinncassiol A glucoside | ADK | 0.1219 |
| P11362 | cinncassiol A glucoside | FGFR1 | 0.1219 |
| Q8IXJ6 | cinncassiol A glucoside | SIRT2 | 0.1219 |
| Q06187 | cinncassiol A glucoside | BTK | 0.1219 |
| P29275 | cinncassiol A glucoside | ADORA2B | 0.1219 |
| P21731 | cinncassiol A glucoside | TBXA2R | 0.1219 |
| P09038 | cinncassiol B glucoside | FGF2 | 0.1219 |
| P17931 | cinncassiol B glucoside | LGALS3 | 0.1219 |
| P56470 | cinncassiol B glucoside | LGALS4 | 0.1219 |
| O00214 | cinncassiol B glucoside | LGALS8 | 0.1219 |
| P51449 | cinncassiol B glucoside | RORC | 0.1219 |
| P06493 | cinncassiol B glucoside | CDK1 | 0.1219 |
| P08913 | cinncassiol B glucoside | ADRA2A | 0.1219 |
| P18825 | cinncassiol B glucoside | ADRA2C | 0.1219 |
| P18089 | cinncassiol B glucoside | ADRA2B | 0.1219 |
| P25100 | cinncassiol B glucoside | ADRA1D | 0.1219 |
| P28223 | cinncassiol B glucoside | HTR2A | 0.1219 |
| P28335 | cinncassiol B glucoside | HTR2C | 0.1219 |
| P35462 | cinncassiol B glucoside | DRD3 | 0.1219 |
| P10635 | cinncassiol B glucoside | CYP2D6 | 0.1219 |
| P50406 | cinncassiol B glucoside | HTR6 | 0.1219 |
| P35348 | cinncassiol B glucoside | ADRA1A | 0.1219 |
| P28222 | cinncassiol B glucoside | HTR1B | 0.1219 |
| Q99808 | cinncassiol B glucoside | SLC29A1 | 0.1219 |
| P11387 | cinncassiol B glucoside | TOP1 | 0.1219 |
| P29274 | cinncassiol B glucoside | ADORA2A | 0.1219 |
| P40763 | cinncassiol B glucoside | STAT3 | 0.1219 |
| P30542 | cinncassiol B glucoside | ADORA1 | 0.1219 |
| P00519 | cinncassiol B glucoside | ABL1 | 0.1219 |
| P29317 | cinncassiol B glucoside | EPHA2 | 0.1219 |
| P06239 | cinncassiol B glucoside | LCK | 0.1219 |
| P12931 | cinncassiol B glucoside | SRC | 0.1219 |
| P80192 | cinncassiol B glucoside | MAP3K9 | 0.1219 |
| P11362 | cinncassiol B glucoside | FGFR1 | 0.1219 |
| O14965 | cinncassiol B glucoside | AURKA | 0.1219 |
| Q06187 | cinncassiol B glucoside | BTK | 0.1219 |
| P0DMS8 | cinncassiol B glucoside | ADORA3 | 0.1219 |
| P00533 | cinncassiol B glucoside | EGFR | 0.1219 |
| P34913 | cinncassiol B glucoside | EPHX2 | 0.1219 |
| P34995 | cinncassiol B glucoside | PTGER1 | 0.1219 |
| P35408 | cinncassiol B glucoside | PTGER4 | 0.1219 |
| P43116 | cinncassiol B glucoside | PTGER2 | 0.1219 |
| P43088 | cinncassiol B glucoside | PTGFR | 0.1219 |
| P43119 | cinncassiol B glucoside | PTGIR | 0.1219 |
| P43115 | cinncassiol B glucoside | PTGER3 | 0.1219 |
| P24557 | cinncassiol B glucoside | TBXAS1 | 0.1219 |
| Q9UQ49 | cinncassiol B glucoside | NEU3 | 0.1219 |
| Q8WWR8 | cinncassiol B glucoside | NEU4 | 0.1219 |
| Q16875 | tangeretin | PFKFB3 | 0.1213 |
| O14746 | tangeretin | TERT | 0.1213 |
| P08183 | tangeretin | ABCB1 | 0.1213 |
| P09917 | tangeretin | ALOX5 | 0.1213 |
| P40763 | cinnzeylanol | STAT3 | 0.1213 |
| P28062 | limonin | PSMB8 | 0.1202 |
| P28074 | limonin | PSMB5 | 0.1202 |
| Q5S007 | limonin | LRRK2 | 0.1202 |
| P42574 | limonin | CASP3 | 0.1202 |
| P55210 | limonin | CASP7 | 0.1202 |
| P56373 | limonin | P2RX3 | 0.1202 |
| P06737 | limonin | PYGL | 0.1202 |
| P28482 | limonin | MAPK1 | 0.1202 |
| P20309 | limonin | CHRM3 | 0.1202 |
| Q02750 | limonin | MAP2K1 | 0.1202 |
| P42345 | limonin | MTOR | 0.1202 |
| P42336 | limonin | PIK3CA | 0.1202 |
| P49810 | limonin | PSEN2 | 0.1202 |
| P48147 | limonin | PREP | 0.1202 |
| Q00987 | limonin | MDM2 | 0.1202 |
| P43235 | limonin | CTSK | 0.1202 |
| P25774 | limonin | CTSS | 0.1202 |
| P29466 | limonin | CASP1 | 0.1202 |
| O00329 | limonin | PIK3CD | 0.1202 |
| Q13547 | limonin | HDAC1 | 0.1202 |
| P56524 | limonin | HDAC4 | 0.1202 |
| P00746 | limonin | CFD | 0.1202 |
| P49841 | limonin | GSK3B | 0.1202 |
| P49840 | limonin | GSK3A | 0.1202 |
| P07711 | limonin | CTSL | 0.1202 |
| P30559 | limonin | OXTR | 0.1202 |
| P21730 | limonin | C5AR1 | 0.1202 |
| P04035 | limonin | HMGCR | 0.1202 |
| P09619 | limonin | PDGFRB | 0.1202 |
| P10721 | limonin | KIT | 0.1202 |
| Q13627 | limonin | DYRK1A | 0.1202 |
| Q16539 | limonin | MAPK14 | 0.1202 |
| P35968 | limonin | KDR | 0.1202 |
| Q15759 | limonin | MAPK11 | 0.1202 |
| Q9HAZ1 | limonin | CLK4 | 0.1202 |
| P49759 | limonin | CLK1 | 0.1202 |
| P49760 | limonin | CLK2 | 0.1202 |
| P56817 | limonin | BACE1 | 0.1202 |
| Q9Y463 | limonin | DYRK1B | 0.1202 |
| P24864 | limonin | CCNE1 | 0.1202 |
| O15055 | limonin | PER2 | 0.1202 |
| P28845 | limonin | HSD11B1 | 0.1202 |
| P56704 | limonin | WNT3A | 0.1202 |
| Q9UGN5 | limonin | PARP2 | 0.1202 |
| Q9H2K2 | limonin | TNKS2 | 0.1202 |
| Q99572 | limonin | P2RX7 | 0.1202 |
| P14416 | limonin | DRD2 | 0.1202 |
| P32246 | limonin | CCR1 | 0.1202 |
| P50750 | limonin | CDK9 | 0.1202 |
| P10275 | limonin | AR | 0.1202 |
| P04150 | limonin | NR3C1 | 0.1202 |
| P14780 | limonin | MMP9 | 0.1202 |
| P29371 | limonin | TACR3 | 0.1202 |
| Q12884 | limonin | FAP | 0.1202 |
| P00734 | limonin | F2 | 0.1202 |
| P06401 | limonin | PGR | 0.1202 |
| P00742 | limonin | F10 | 0.1202 |
| P34972 | limonin | CNR2 | 0.1202 |
| P03956 | limonin | MMP1 | 0.1202 |
| P31749 | limonin | AKT1 | 0.1202 |
| Q9NWZ3 | limonin | IRAK4 | 0.1202 |
| O14684 | limonin | PTGES | 0.1202 |
| P29275 | limonin | ADORA2B | 0.1202 |
| P42338 | limonin | PIK3CB | 0.1202 |
| O14965 | limonin | AURKA | 0.1202 |

Table S4. Mass spectrometry information of differential metabolites.

| No. | RT (min) | Metabolites | Formula | ΔPPM^a^ | Adduct | Detected MS (m/z)^b^ | MS/MS(m/z)^c^ |
| --- | --- | --- | --- | --- | --- | --- | --- |
| 1 | 16.91 | Ubiquinone-2 | C_19_H_26_O_4_ | 9.09 | M+H | 319.1938 | 287.17,201.13,123.12 |
| 2 | 16.4 | n6-[2-(4-Aminophenyl)ethyl]adenosine | C_18_H_22_N_6_O_4_ | 3.91 | M+Na | 409.1616 | 387.17,253.12,150.08 |
| 3 | 16.41 | Dodec-7-enedioylcarnitine | C_19_H_33_NO_6_ | -6.39 | M+H | 313.1631 | 144.10 |
| 4 | 16.19 | Stearoylcarnitine | C_25_H_49_NO_4_ | 1.87 | M+H | 428.3748 | 369.30,144.11 |
| 5 | 15.23 | Hydroxyeicosatetraenic acid | C_20_H_32_O_3_ | -7.16 | M+H | 321.2407 | 289.22,275.21,261.18 |
| 6 | 14.74 | Hydroxylinolenic acid | C_18_H_30_O_3_ | -3.78 | M+Na | 317.2081 | 147.12,131.09,103.05 |
| 7 | 16.01 | Octadecatetraenoic acid | C_18_H_28_O_2_ | -3.97 | M+H | 277.2157 | 133.10,119.09,105.07 |
| 8 | 16.49 | Cosahexaenoic acid | C_20_H_28_O_2_ | -0.62 | M+Na | 323.1985 | 287.17,201.13,123.12 |
| 9 | 15.31 | Sphinganine | C_18_H_39_NO_2_ | 1.98 | M+H | 302.3065 | 254.29,109.10 |
| 10 | 17.47 | Cer (t18:0/16:0) | C_34_H_69_NO_4_ | 4.49 | M+H | 556.5330 | 300.29,120.07,148.09 |
| 11 | 16.23 | Ganglioside GA2 (d18:1/9Z-18:1) | C_56_H_102_N_2_O_18_ | -2.82 | M+Na | 1114.6786 | 309.30,162.08 |
| 12 | 16.46 | Ganglioside GM3 (d18:0/12:0) | C_53_H_98_N_2_O_21_ | 11.41 | M+Na | 1121.6688 | 945.50,300.29 |
| 13 | 18.09 | DG (PGD1/i-15:0) | C_38_H_68_O_8_ | 2.3 | M+H | 653.5007 | 413.29,145.08,119.08 |
| 14 | 16.48 | PS (20:4/18:0-2OH) | C_44_H_78_NO_12_P | 5.21 | M+H | 844.5384 | 339.29 |
| 15 | 16.48 | PC (LTE4/22:0) | C_53_H_97_N_2_O_11_PS | -11.04 | M+Na | 1023.6335 | 184.07,125.00 |
| 16 | 16.84 | PE (20:4-2OH/DiMe) | C_47_H_80_NO_11_P | 3.69 | M+H | 866.5579 | 506.29,339.29 |
| 17 | 16.48 | PG (LTE4/20:2) | C_49_H_84_NO_13_PS | -0.1 | M+Na | 980.5298 | 956.54,574.23 |
| 18 | 16.81 | PG (i-14:0/i-13:0) | C_33_H_65_O_10_P | 7.85 | M+Na | 675.4266 | 481.43,255.23,155.18 |
| 19 | 17.65 | LysoPI (20:4) | C_29_H_49_O_12_P | 11.5 | M+Na | 643.2933 | 361.27,259.25,110.98 |
| 20 | 15.88 | LysoPE (22:6) | C_27_H_44_NO_7_P | 0 | M+Na | 548.2753 | 171.12,142.03 |
| 21 | 16.16 | LysoPE (22:5) | C_27_H_46_NO_7_P | -2.08 | M+H | 528.3079 | 313.27 |
| 22 | 17.06 | LysoPE (18:0/0:0) | C_23_H_48_NO_7_P | 2.07 | M+H | 482.3257 | 313.27,393.25 |
| 23 | 16.33 | LysoPE (16:0) | C_21_H_44_NO_7_P | 0.88 | M+H | 454.2938 | 313.27 |
| 24 | 16.88 | LysoPC (P-18:0) | C_26_H_54_NO_6_P | -5.28 | M+Na | 530.3558 | 144.10 |
| 25 | 17.45 | LysoPC (O-18:0) | C_26_H_56_NO_6_P | -0.39 | M+H | 510.3922 | 184.07,125,104.11 |
| 26 | 16.46 | LysoPC (18:1) | C_26_H_52_NO_7_P | -3.93 | M+K | 560.3096 | 184.07,125,104.11 |
| 27 | 16.68 | LysoPC (20:2) | C_28_H_54_NO_7_P | -3.51 | M+Na | 570.3516 | 184.07,125.00 |
| 28 | 15.99 | Dihydrotestosterone diglucuronide | C_31_H_46_O_14_ | -11.57 | M+Na | 665.2708 | 233.19,177.03 |
| 29 | 18.59 | PGD2 ethanolamide | C_22_H_37_NO_5_ | 7.67 | M+H-H_2_O | 378.2673 | 144.10 |
| 30 | 16.66 | LTB4 ethanolamide | C_22_H_37_NO_4_ | 5.97 | M+Na | 402.2644 | 344.26,301.21,137.09 |
| 31 | 16.81 | Arachidonic acid | C_20_H_32_O_2_ | 2.62 | M+H | 305.2489 | 245.2264/184.1463/107.0852 |
| 32 | 15.31 | Sulfolithocholylglycine | C_26_H_43_NO_7_S | -3.31 | M+H | 514.2821 | 412.2846/388.2822/123.1123 |

^a^ The measured m/z values minus the theoretical values that acquired by using HMDB and METIN.

^b^ The measured m/z values by MS^E^ analysis.

^c^ The values of fragment ions.
